# Immune Landscape and an RBM38-Associated Immune Prognostic Model with Laboratory Verification in Malignant Melanoma

**DOI:** 10.3390/cancers14061590

**Published:** 2022-03-21

**Authors:** Jinfang Liu, Jun Xu, Binlin Luo, Jian Tang, Zuoqiong Hou, Zhechen Zhu, Lingjun Zhu, Gang Yao, Chujun Li

**Affiliations:** 1 Department of Plastic and Burns Surgery, The First Affiliated Hospital of Nanjing Medical University, 300 GuangZhou Rd, Nanjing 210029, China; liujinfang55555@163.com (J.L.); luobin8212192340@sina.com (B.L.); tangjian@njmu.edu.cn (J.T.); houzuoqiong@jsph.org.cn (Z.H.); zhuzc3000@126.com (Z.Z.); 2 Department of Oncology, The Third Affiliated Hospital of Soochow University, Soochow 213000, China; xujunloloxj@163.com; 3 Department of Oncology, The First Affiliated Hospital of Nanjing Medical University, Nanjing 210029, China; lingjunzhu233@163.com

**Keywords:** melanoma, RBM38, prognostic signature, tumor microenvironment, immune checkpoint therapy

## Abstract

**Simple Summary:**

The primary treatment of malignant melanoma is a classical regimen of surgery combined with chemotherapy, targeted drugs, and immunotherapy. The purpose of this study was to explore the immune response mechanism of RNA binding protein RBM38 in the development of melanoma with the screening of effective immunodiagnostic models and targeted therapy. We found that RBM38, as an oncogene, promotes the proliferation, invasion, and migration of melanoma cells and is associated with immune infiltration and pathways. Our investigation presented the prognostic significance of RBM38-associated immune signature. In addition, this model may provide a potential strategy for improving the survival and immunotherapy of melanoma patients.

**Abstract:**

Background: Current studies have revealed that RNA-binding protein RBM38 is closely related to tumor development, while its role in malignant melanoma remains unclear. Therefore, this research aimed to investigate the function of RBM38 in melanoma and the prognosis of the disease. Methods: Functional experiments (CCK-8 assay, cell colony formation, transwell cell migration/invasion experiment, wound healing assay, nude mouse tumor formation, and immunohistochemical analysis) were applied to evaluate the role of RBM38 in malignant melanoma. Immune-associated differentially expressed genes (DEGs) on RBM38 related immune pathways were comprehensively analyzed based on RNA sequencing results. Results: We found that high expression of RBM38 promoted melanoma cell proliferation, invasion, and migration, and RBM38 was associated with immune infiltration. Then, a five-gene (A2M, NAMPT, LIF, EBI3, and ERAP1) model of RBM38-associated immune DEGs was constructed and validated. Our signature showed superior prognosis capacity compared with other melanoma prognostic signatures. Moreover, the risk score of our signature was connected with the infiltration of immune cells, immune-regulatory proteins, and immunophenoscore in melanoma. Conclusions: We constructed an immune prognosis model using RBM38-related immune DEGs that may help evaluate melanoma patient prognosis and immunotherapy modalities.

## 1. Introduction

Malignant melanoma is an exceedingly invasive and easily metastasizing form of skin cancer. It has an annual growth rate of 3 to 5%, making it the fastest-growing of all cancer malignancies and a global public health risk [1]. Meanwhile, the long-term survival of malignant melanoma patients remains extraordinarily low due to recurrence and metastasis [2]. The primary treatment method for malignant melanoma is the classical plan of surgery combined with chemotherapy [3], targeted medicines, and immunotherapy [4,5]. Immunotherapy, a promising treatment method, activates the immune system to fight against cancer cells; conversely, preventing immunity is crucial for cancer cell survival. Immunity can be achieved by manipulating immune checkpoints, which are often used to maintain self-tolerance. Emerging reports show that excellent results have been achieved with immune checkpoint inhibitors (ICIs) in immunotherapy for advanced melanoma patients, and they open new possibilities for the treatment of malignant melanoma [6,7].

RNA-binding protein (RBP) is a type of protein that has a high affinity for mRNA and can promote mRNA degradation or enhance its stability by binding to cis-regulatory elements (CREs) [8]. Different RBPs can have tumor-suppressive or oncogenic functions in human cancers [9]. RBM38 (alias RNPC1) is an RBP that includes putative RNA recognition motifs (RRMs) and shares the same Hu antigen R (HuR) and Musashi antigen sequences [10]. Recent studies have shown that RBM38 is oncogenic and amplified in chronic lymphocytic leukemia [11], ovarian cancer [12], prostate cancer [13,14], etc. Conversely, some studies have demonstrated that RBM38 has a tumor-suppressive effect in colorectal cancer [15], breast cancer [16], non-small cell lung cancer [17], esophageal adenocarcinoma [18], etc. These contradictory results indicate that RBM38 can promote or suppress cancer progression in different tumors by regulating different signaling pathways. The expression and biological function of RBM38 in malignant melanoma has not been analyzed, and it might provide a relevant diagnostic and therapeutic tool in malignant melanoma.

The poor prognosis for melanoma patients highlights the urgent need for more prospective immune biomarkers and better alternative therapeutic strategies for this aggressive disease. In this research, we evaluated the role of RBM38 in malignant melanoma and formulated an RBM38-associated immune prognostic model (RAIPM) that can estimate the prognosis for melanoma patients.

## 2. Materials and Methods

### 2.1. RBM38 Expression in Different Datasets

The gene expression profiles and clinical information of malignant melanoma patients were collected from The Cancer Genome Atlas (TCGA; https://portal.gdc.cancer.gov (accessed on 15 September 2021)) and Gene Expression Omnibus (GEO; https://www.ncbi.nlm.nih.gov/geo (accessed on 15 September 2021)) datasets. The normal expression profiles of the control gene were duplicated from the Genotype–Tissue Expression (GTEx; https://www.gtexportal.org (accessed on 15 September 2021)) dataset. The gene expression profiles in the TCGA-SKCM and GTEx-skin datasets were analyzed using the same sequencing platform and library preparation to reduce potential batch effects [19].

### 2.2. Survival Prognosis and Genetic Alteration of RBM38

A pan-cancer analysis of RBM38 was performed with SangerBox (http://sangerbox.com/Tool (accessed on 15 September 2021)). The TCGA-SKCM and GSE65904 melanoma samples with complete expression pro-files and clinical information were used to construct and verify the prognostic model. In addition, the GSE22155 dataset was used to analyze the survival of RBM38. The genetic alteration characteristics of RBM38 in melanoma were explored in cBioPortal (https://www.cbioportal.org/ (accessed on 15 September 2021)).

### 2.3. Cell Culture and Transfection

Human malignant melanoma A375 and M14 cell lines were acquired from the Chinese Academy of Sciences and Cell Resource Center of Shanghai Life Sciences Institute. We placed A375 cells in RPMI 1640 medium and added 10% fetal bovine serum (FBS), 1% streptomycin and penicillin (Gibco, Invitrogen, Waltham, MA, USA). M14 cells were cultured in a DMEM medium containing 1% antibiotics and 15% FBS. RBM38 overexpression and knockdown were generated using lentivirus constructs (GenePharma, Suzhou, China). In addition, RBM38 shRNA sequences were listed in Table S1. Briefly, cells were seeded at 50% confluence in a 6-well plate and infected with retroviruses (scramble control (Ctrl), RBM38 overexpression (RBM38), negative control (NC), and knockdown (shRBM38-2). Stable transduction of RBM38 was achieved by puromycin (5 µg/mL) selection.

### 2.4. Real-Time Quantitative RT-PCR (qRT-PCR)

As previously presented [20], Total RNAs were extracted from melanoma cells and tissues using the Trizol reagent (Invitrogen, Waltham, MA, USA), and cDNA was then synthesized using Primescript RT Reagent (Takara, Kusatsu, Japan) according to the manufacturer’s instructions. qRT-PCR was carried out on StepOnePlus Real-Time PCR system (Applied Biosystems, Waltham, MA, USA) or LightCycler 480 (Roche, South San Francisco, CA, USA) with the SYBR qPCR Master Mix (Vazyme Biotechnology, Q311-02/03 or Q712-02, Nanjing, China). All samples were detected in triplicate. The data were analyzed by the 2^−ΔΔCt^ method. The primers are listed in Table S2.

### 2.5. CCK-8 and Cell Colony Assay

Cell proliferation of A375 and M14 cells were executed via Cell Counting Kit 8 (CCK-8) assay (Obio Technology Corp, Shanghai, China) and cell colony formation according to the manufacturer’s protocol, as in our recent report [20].

### 2.6. Xenograft Tumor in Nude Mice

The experiments with BALB/c nude mice were conducted strictly based on the guidelines of the Institutional Animal Care and Use Committee of the Nanjing Medical University. We purchased female nude mice (4–6 weeks, 18–22 g) from the Model Animal Research Center of Nanjing University (Nanjing, China). They were then randomly divided into 4 groups: RBM38, Ctrl, shRBM38, and NC. Stably transfected A375 cells (1 × 10^6^ cells in 100 µL PBS volume) were subcutaneously injected into the left flank of each mouse. The growth of tumors and mice was measured for 18 days. Tumor volume was calculated every other day with Vernier calipers according to the formula (length × width^2^)/2.

### 2.7. Transwell Cell Migration and Invasion Experiment

A375 and M14 cells were cultured with 50 mL of Matrigel (BD Biosciences, Franklin Lakes, NJ, USA) through 24-well transwell inserts (Millicell hanging cell culture insert, Millipore, Billerica, MA, USA). Inserts were seeded with 5 × 10^4^ cells with 0.1% FBS supplemented with 200 uL DMEM and a complete medium (500 uL) with 10% FBS was placed in the chamber underneath. Cells for migration experiments were similarly precoated without Matrigel. Non-invading cells on the upper side of the membranes were cleaned after incubation for 36 h at 37 °C. According to the manufacturer's instructions, we used the Hema 3 statistics pack (Fisher Scientific, Hampton, NH, USA) for manual staining to fix and stain invading or migrating cells attached to the outside of the cell culture insert. Then we counted the number of invaded or migrated cells in 5 independent fields under the microscope. The graph shows the average number of cells in each field. In our recent study, we performed an M14 cell wound healing assay [20].

### 2.8. Immunohistochemical Staining

The dewaxed slices were placed in 0.01 M citrate buffer to collect the antigen. After blocking endogenous peroxidase activity in 3% H_2_O_2_, the slices were incubated with RBM38, CD11b, CD19, and E-cadherin antibody (species, clone, and catalog number of antibodies are listed in Table S2) at a dilution of 1:100 at 4 °C overnight. Then the slices were rinsed, and the Real Envision Detection Kit (GeneTech, Shanghai, China) was used to visualize immunoperoxidase staining according to the manufacturer’s instructions. The immunohistochemical (IHC) staining score was evaluated in a semi-quantitative method combining the intensity and distribution of specific staining by two pathologists. The staining intensity was graded from 0 to 3, and the staining percentage of positive cells was classified into four grades (percentage score 1–4).

### 2.9. RNA Sequencing Analysis

One sample each of 3 × 10^6^ well-conditioned RBM38 overexpression (RBM38) and control (NC) A375 cells was utilized for total RNA isolation. Subsequently, transcriptome sequencing was carried out on the Illumina HiSeq 4000 platform by the Beijing Allwegene Technology Co., Ltd. (Beijing, China). Finally, TopHat2 software (v2.1.0) was used to map the clean reads with the reference genome. Differentially expressed genes (DEGs) (FPKM > 1, *p* < 0.05) were found by DESeq (1.10.1).

### 2.10. GO and KEGG Analyses

The expression profiles of DEGs from the RNA sequencing analysis were utilized to analyze the difference by Gene Ontology (GO) enrichment and Kyoto Encyclopedia of Genes and Genomes (KEGG) pathway analysis.

### 2.11. Selection and Analysis of Immune-Related Differential Expressed Genes

The Limma package screened out genes enriched on the immune path-ways from the GO and KEGG analysis. Next, the pheatmap package was used to delineate immune-associated DEGs.

### 2.12. Construction and Validation of Prognostic Models

Univariate Cox regression analysis was conducted to analyze the prognosis value of the DEGs in the training cohort. DEGs associated with overall survival (OS) in univariate analysis were subsequently chosen to build a prognostic model by the LASSO Cox regression algorithm [21]. Then the optimal penalty parameter associated with the minimum ten-fold cross-validation was selected. Finally, the risk score of the signature was equal to the result of (λ1 × expression of A) + (λ2 × expression of B) + (λ3 × expression of C) + … + (λn × expression of N), where λ indicates the coefficient of regression for each gene.

### 2.13. Independent Prognostic Ability of the Five-Gene Signature

It is important to determine whether the RAIPM risk score of clinicopathologic characteristics (AJCC-TNM stage, age, and gender) were independent prognostic factors for patients with melanoma; thus, univariate and multivariate Cox regression analyses were carried out on the validation and training cohorts. The Kaplan–Meier method was used to identify differences in OS between high-risk and low-risk subgroups, stratified by age, gender, and AJCC staging. The AUC value of the ROC curve was generated using the survival and timeROC packages in R.

### 2.14. Immune Infiltration Analysis

The GSVA package was used to explore immune infiltration. Next, differences in the composition of immune cell infiltration were analyzed between low- and high-risk groups. Correlation analysis between risk scores of our five-gene signature and infiltration of immune cells was visualized through the ggplot2 package.

### 2.15. Immunophenoscore

Immunophenoscore (IPS), collected from the Cancer Immunome Atlas dataset (https://www.tcia.at (accessed on 17 September 2021)), was utilized to assess tumor immunogenicity. The analysis and calculation of IPS used four main categories of machine learning genes to identify immunity (MHC molecular effector cells, immunosuppressive cells, immunomodulators). IPS is calculated based on the Z-scores of gene expression from representative cell types (e.g., CD4^+^ T cells activation, CD8^+^ T cells, CD4^+^ T cells effect of the activation of memory cells, CD4^+^ T cells, subpopulations, MDSCs) in the range of 0–10. Higher values reflect increased immunogenicity.

### 2.16. Statistical Analysis

Except for the above-mentioned R software analysis (version 4.0.1; https://www.r-project.org (accessed on 19 May 2020)), other statistical data were analyzed using GraphPad Prism 8.0 (GraphPad Software, San Diego, CA, USA) and SPSS 26.0 (IBM, Armonk, NY, USA). Data are shown as the mean ± SD of at least three replicates. Rational statistical tests were conducted between two independent experimental sets (Student’s *t*-test) and among more than two experimental sets (ANOVA test). *p* < 0.05 indicates statistically significant differences.

## 3. Results

### 3.1. RBM38 Expression, Alteration, and Prognosis Analysis

**Figure 1:**
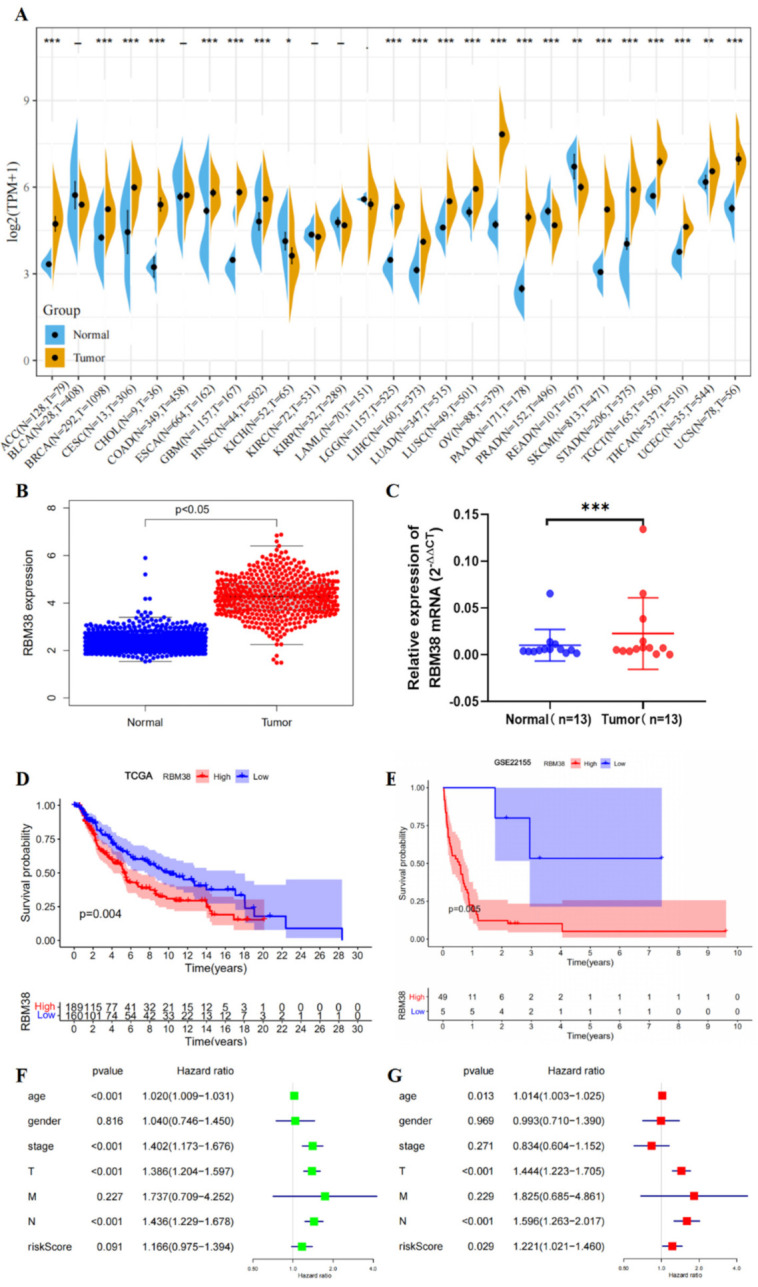
Expression level of RBM38 gene in different tumors and OS in melanoma patients. (**A**) The expression status of the RBM38 gene in various types of cancers in TCGA and GTEx databases. (* *p* < 0.05; ** *p* < 0.01; *** *p* < 0.001). (**B**) For melanoma in the TCGA project, the corresponding normal tissues of the GTEx-skin dataset served as controls. (**C**) RT-qPCR analysis of RBM38 expression of mRNA in 13 pairs of melanoma tissues and matched normal tissues quantified after transfection. (Data are shown as the mean ± SD of three replicates. *** *p* < 0.001 by ANOVA test). (**D**,**E**) The TCGA samples with entire survival and clinical information and the GSE22155 samples were used to analyze the survival of RBM38. (**F**,**G**) Univariate and multivariate Cox analyses of the expression of RBM38 and clinicopathological parameters in the TCGA group with complete survival and clinical information.

First, we explored the role of RBM38 in malignant melanoma. We assessed the differences in RBM38 expression between normal tissue and tumor samples of 27 cancers with the TCGA and GTEx datasets. RBM38 was overexpressed in 19 types of cancer samples, including skin cutaneous melanoma (SKCM) (Figure 1A,B). Meanwhile, RT-qPCR analysis of RBM38 mRNA expression indicated that RBM38 level was higher in 13 paired melanoma tissues than matched normal tissues (Figure 1C). Based on the expression levels of RBM38, research cases were divided into low- and high-expression cohorts. Melanoma patients with low-expression RBM38 were linked to better prognosis in the TCGA and GSE22155 datasets (Figure 1D,E).

The type, site, and case number of the RBM38 genetic alteration were further illustrated in Figure S1A. The genetic alteration status of RBM38 showed that the “amplification” type of CNA was in the metastatic melanoma (Cell 2016; Science 2015), melanoma (NEJM 2014), and SKCM (TCGA, PanCancer Atlas), which showed an alteration frequency from 1% to 20% (Figure S1B). We explored the RBM38 expression in the TCGA mutation database. However, only four samples were in the mutation group and 363 not mutated group (Figure S1C). Therefore, the correlation analysis between RBM38 expression and mutation types was inefficient. Furthermore, the survival probability analysis was not efficient (Figure S1D). Univariate Cox analysis demonstrated that high RBM38 expression predicted worse OS (HR = 1.166, *p* = 0.091) (Figure 1F). Multivariate Cox analysis was subsequently used to assess the variables in the previous analysis. The results showed that RBM38 was identified as an independent factor for poor OS in patients with malignant melanoma (HR = 1.221, *p* = 0.029) (Figure 1G).

### 3.2. RBM38 Promotes Melanoma Cell Proliferation, Migration, and Invasion and Influences Immune Cell Infiltration

**Figure 2:**
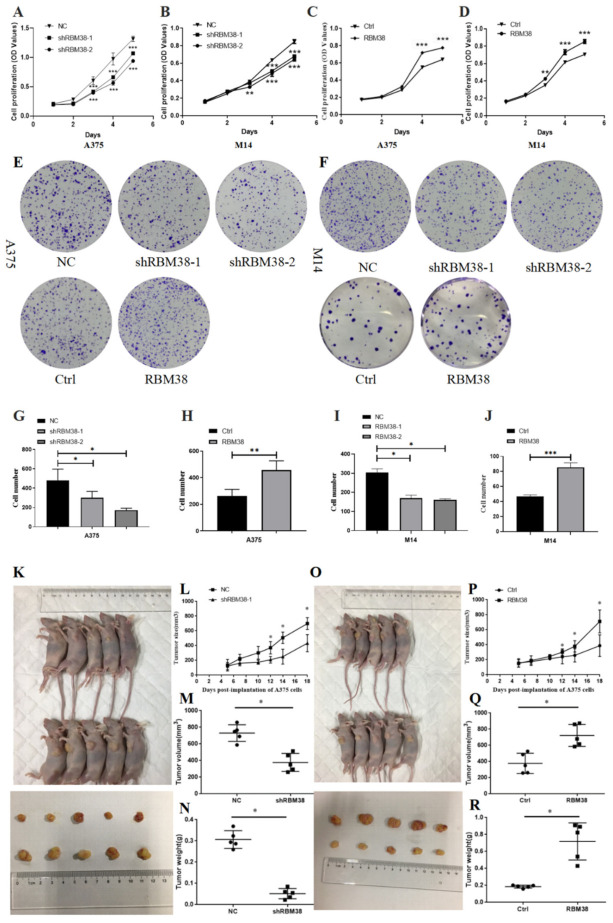
RBM38 promoted melanoma cell proliferation in vitro and vivo. (**A**–**D**) In A375 and M14 cells, the CCK-8 assay exhibited the effect of overexpression and downregulation of RBM38 on cell proliferation (data are shown as the mean ± SD of three replicates). (**E**–**J**) In A375 and M14 cells, colony formation assays exhibited the effect of overexpression and downregulation of RBM38 on cell proliferation. (Data are shown as the mean ± SD of three replicates). (**K**–**N**) Knockdown of RBM38 expression significantly inhibited melanoma cancer cell growth in nude mice and tumor volume weight was significantly reduced in the sh-RBM38 group compared to that in the NC group (there are five mouse tumors in each group). (**O**–**R**) Overexpression of RBM38 expression significantly increased melanoma cancer cell growth in nude mice and tumor volume weight significantly increased in the RBM38 group compared to that in the Ctrl group (there are five mouse tumors in each group) (for statistical comparison between two independent experimental groups (Student’s *t*-test) and among more than two experimental groups (ANOVA test), appropriated statistical tests were assayed. * *p* < 0.05, ** *p* < 0.01, *** *p* < 0.001).

**Figure 3:**
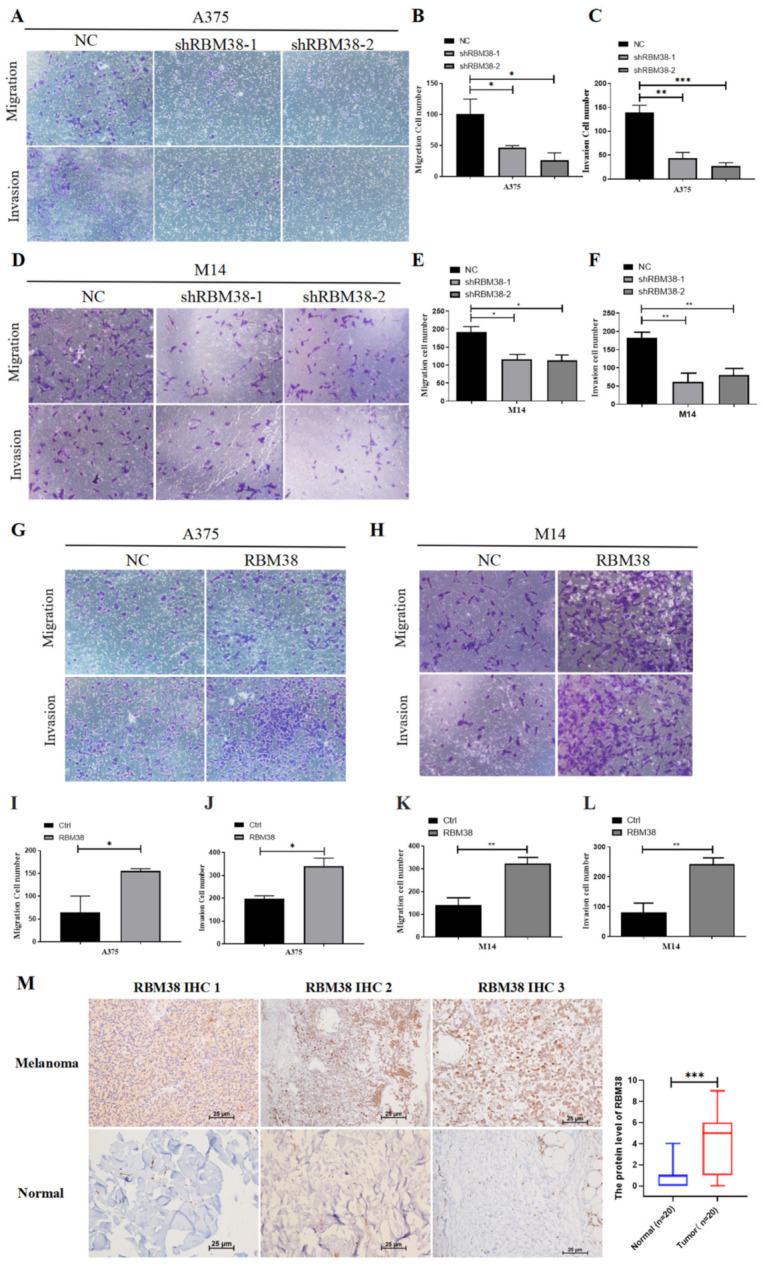
RBM38 promoted melanoma cell migration in vitro and in vivo and is highly expressed in melanoma tissues. (**A**–**F**) Effects of RBM38 knockdown on cell invasion and migration by cell invasion and migration assay in A375 and M14 cells (data are shown as the mean ± SD of three replicates). (**G**–**L**) Effects of RBM38 overexpression on cell invasion and migration by cell invasion and migration assay in A375 and M14 cells (data are shown as the mean ± SD of three replicates). (**M**) Representative images and statistical graphs of RBM38 staining in melanoma and normal tissues (*n* = 20, *** *p* < 0.001) by IHC staining. Scale bars indicated 25 μm. (For statistical comparison between two independent experimental groups (Student’s *t*-test) and among more than two experimental groups (ANOVA test), appropriated statistical tests were assayed. * *p* < 0.05, ** *p* < 0.01, *** *p* < 0.001).

The qRT-PCR analysis initially demonstrated that RBM38 in A375 and M14 cells were steadily knocked down and overexpressed (Figure S2A–D). The effects of RBM38 on cell proliferation and inhibition were subsequently detected in A375 and M14 cells. In CCK-8 and colony formation, knockdown of RBM38 remarkably inhibited cell proliferation, while overexpression promoted cell proliferation (Figure 2A–J). Moreover, in vivo tumor formation experiments showed that knockdown of RBM38 expression significantly inhibited melanoma cancer cell growth (Figure 2K–N), and overexpression of RBM38 expression significantly increased melanoma cancer cell growth in nude mice (Figure 2O–R). In addition, cell migration and invasion were inhibited by RBM38 knockdown and expanded by RBM38 overexpression through transwell cell migration and invasion experiments in A375 and M14 cells (Figure 3A–L) and wound healing assay in M14 cells (Figure S2E–H). IHC analysis with staining E-cadherin was performed (Figure S2I,K). In addition, the results showed that RBM38 was positively correlated with E-cadherin. The experiments confirmed that RBM38 could promote invasion and migration in melanoma. Furthermore, IHC analysis provided further evidence that RBM38 protein expression level was higher in melanoma samples than normal tissue (*n* = 20 in each group) (Figure 3M and Figure S3A). In addition, the sections of negative control were not subjected to the primary antibodies (Figure S3B).

### 3.3. RBM38 Influences Immune Cell Infiltration and Promotes the Proliferation and Migration by Immune Pathway

**Figure 4:**
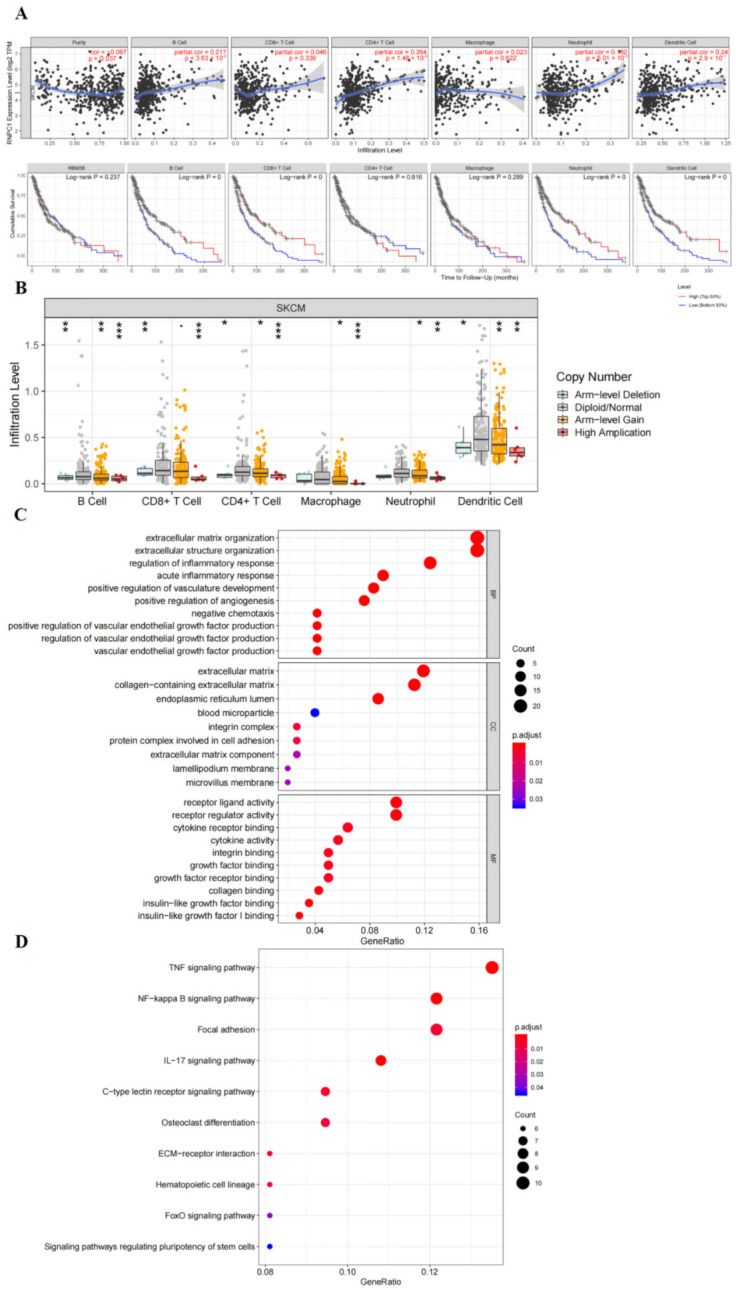
RBM38 correlated with the immune system and GO and KEGG pathway analysis. (**A**) The correlation between RBM38 expression and the level of immune cell infiltration and in the database of TIMER. (**B**) The correlation between RBM38 copy number variation (CNV) and the level of immune cell infiltration in the TIMER database. (* *p* < 0.05, ** *p* < 0.01, *** *p* < 0.001). (**C**) Bubble plots of GO analysis of the biological process of the differentially expressed genes related to RBM38. (**D**) Bubble plots of KEGG analysis of the biological process of the differentially expressed genes related to RBM38.

The association between immune cells and RBM38 expression in malignant melanoma was analyzed with the Tumor Immune Estimation Resource (TIMER; cistrome.shinyapps.io/timer (accessed on 17 September 2021)) database. It was found that RBM38 was related to tumor purity (r = −0.097, *p* = 0.037), B cell infiltration (r = 0.271, *p* < 0.001), CD4^+^ T infiltration (r = 0.264, *p* < 0.001), neutrophil infiltration (r = 0.132, *p* = 0.005), and dendritic cell infiltration (r = 0.24, *p* < 0.001) (Figure 4A). RBM38 CNV was found to be strongly related to immune infiltration in malignant melanoma, containing CD4^+^ T cells, CD8^+^ T cells, B cells, neutrophils, macrophages, and dendritic cells (Figure 4B). These results demonstrate that RBM38 may be correlated with immune cell infiltration in the TME of malignant melanoma. Given this connection, it is relatively evident that the immune pathway may be involved in tumor initiation and progression. Therefore, further detailed experiments were conducted to prove this speculation.

We used Spearman’s correlation analysis to describe the relevance of immune cells in the tumor microenvironment (TME). As indicated in the pheatmap (Figure S4A), the immune cells exhibited intricate relevance, and B cell memory was significantly correlated with the most immune cells. We performed the relevance analysis of immune cells in the TME and the principal component analysis (PCA) analysis of tumor-infiltrating lymphocytes (TiL), tumor-infiltrating myeloid cells (TiM), immune checkpoint block (ICB), and RBM38. The results showed that RBM38 was correlated with ICB through PCA analysis (Figure S4B). Therefore, it is essential to identify further the association between RBM38 expression, immune cells, and ICB therapy.

Previous research showed that CD11b was the cell surface marker of neutrophils [22] and dendritic [23], and CD19 were the marker of B cells [24] and neutrophils [25]. Therefore, we performed protein analysis by IHC staining with CD11b and CD19. Then we included some quantitative analysis in the IHC staining, and the results showed that RBM38 was positively related to CD 11b and CD19 (Figure S2I,J,L,M). It means that B cells, neutrophils, and dendritic cells are concomitantly expressed with RBM38.

RNA sequencing profiling indicated that 158 downregulated genes were differentially expressed in shRBM38 versus NC cells (false discovery rate (FDR) < 0.05; LogFC > 1) (Figure S4C). Next, GO and KEGG analyses of the downregulated DEGs were conducted. The functional annotations by GO analysis indicated the RBM38 related DEGs were involved in the immune-related pathway, including acute inflammatory response, negative chemotaxis, regulation of inflammatory response, cytokine activity, and cytokine receptor binding. The KEGG analysis indicated that RBM38 related DEGs were involved in the NF-kappa B, TNF, and IL-17 signaling pathways (Figure 4C,D).

### 3.4. Construct a Five-Gene Prognostic Signature

**Figure 5:**
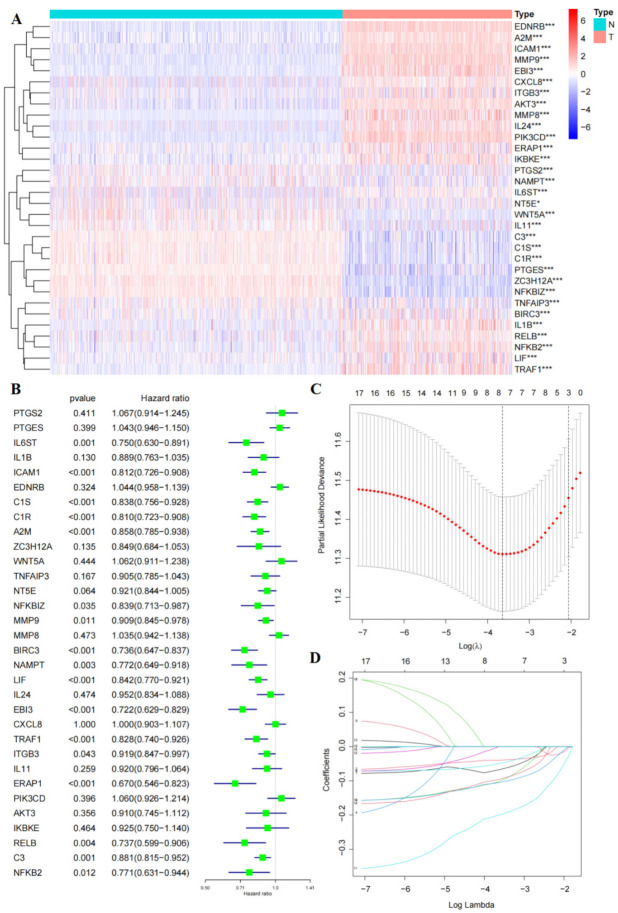
The expression pattern of RBM38-related immune genes in the training cohort and construction of prognostic risk signature. (**A**) The expression pattern of RBM38-related immune genes in the training cohort (* *p* < 0.05, *** *p* < 0.001). (**B**) RBM38-related immune genes were assessed by univariate Cox analysis in the training cohort. (**C**,**D**) LASSO Cox regression analysis of the chosen 17 RBM38-related immune regulators(* *p* < 0.05, *** *p* < 0.001).

A total of 32 immune-related genes were collected from the RBM38 related DEGs enriched in the immune pathway (Figure 5A). An elaborate classification was exerted to describe the distribution of clinical characteristics of malignant melanoma patients using the TCGA-SKCM and GSE65904 datasets, shown in Tables S3 and S4. Then the complete expression profiles and clinical information of the TCGA-SKCM group (*n* = 349) (Table S5) was utilized to establish a training cohort for the prognostic model. In addition, the prognostic model was validated by the GSE65904 group (*n* = 210) (Table S6). Univariate Cox regression analysis indicated that 17 out of 32 immune-related genes were notably related to OS in the training cohort (Figure 5B, *p* < 0.05). LASSO Cox regression analysis could more accurately evaluate the clinical outcomes of patients with melanoma. According to the minimum criteria, five key genes (A2M, NAMPT, LIF, EBI3, and ERAP1) were screened (Figure 5C,D). Next, the five hub genes were subjected to multivariate Cox regression to establish an excellent RBM38-associated immune prognostic model (RAIPM) (Table 1). Using the coefficients obtained by multivariate Cox analysis, the risk score of melanoma patients was calculated according to the following formula: risk score = (−0.097) × A2M + (−0.185) × NAMPT + (−0.123) × LIF + (−0.247) × EBI3 + (−0.170) × ERAP1.

### 3.5. Risk Score of RAIPM Is an Independent Prognostic Factor

**Figure 6:**
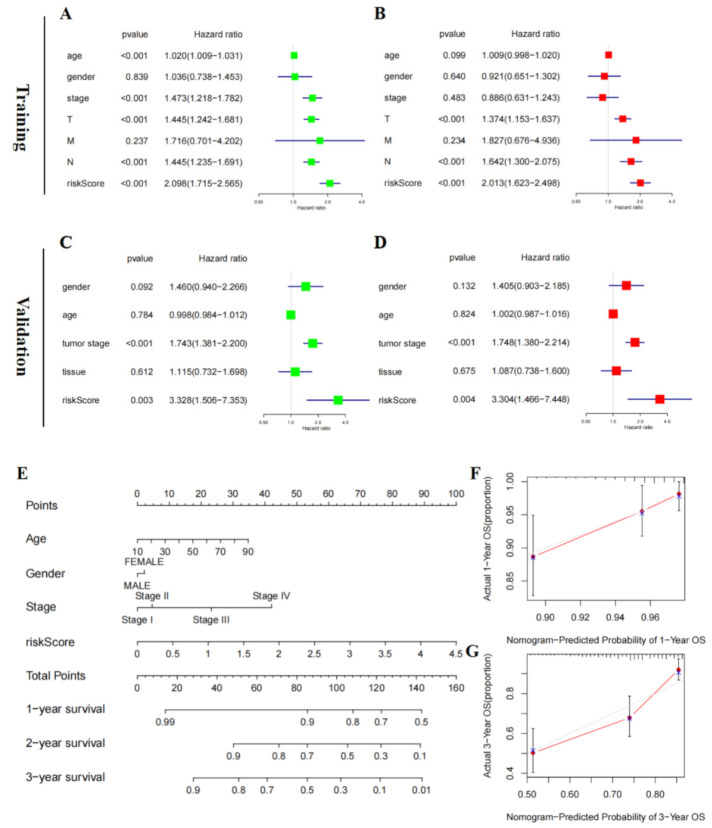
Independent prognostic factors in the validation and training cohorts. (**A**,**B**) Univariate and Multivariate Cox analyses of the risk score of our prognostic signature and clinicopathological parameters in the training cohort. (**C**,**D**) Univariate and Multivariate Cox analyses of the risk score of our prognostic signature clinicopathological parameters in the validation cohort. (**E**) Prognostic nomogram for malignant melanoma patients. (**F**,**G**) Calibration curves for the 1- and 3-year nomogram.

The RAIPM-based risk score was correlated with poor OS according to univariate Cox analysis in the training cohort (HR = 2.098, *p* < 0.001) (Figure 6A). Remarkably, the analysis revealed that the risk score of RAIPM was an independent risk factor for worse OS (HR = 2.013, *p* < 0.001) (Figure 6B). Meanwhile, in the validation cohort, univariate and multivariate analyses showed that a high-risk score (HR = 3.328, *p* = 0.003 and HR = 3.304, *p* = 0.004; Figure 6C,D) was remarkably related to poor OS. Briefly, these results show that the risk score of RAIPM is an independent prognostic factor in malignant melanoma.

A nomogram was developed to predict the prognosis of patients with malignant melanoma in the training cohort. Four clinical prognostic factors of OS (RAIPM risk score, gender, age, and stage) were integrated into the nomogram (Figure 6E). The calibration plots show that the actual observations exactly match the predicted 1- and 3-year OS rates (Figure 6F,G).

### 3.6. Validate the Prognostic Value of the Five-Gene Risk Signature

**Figure 7:**
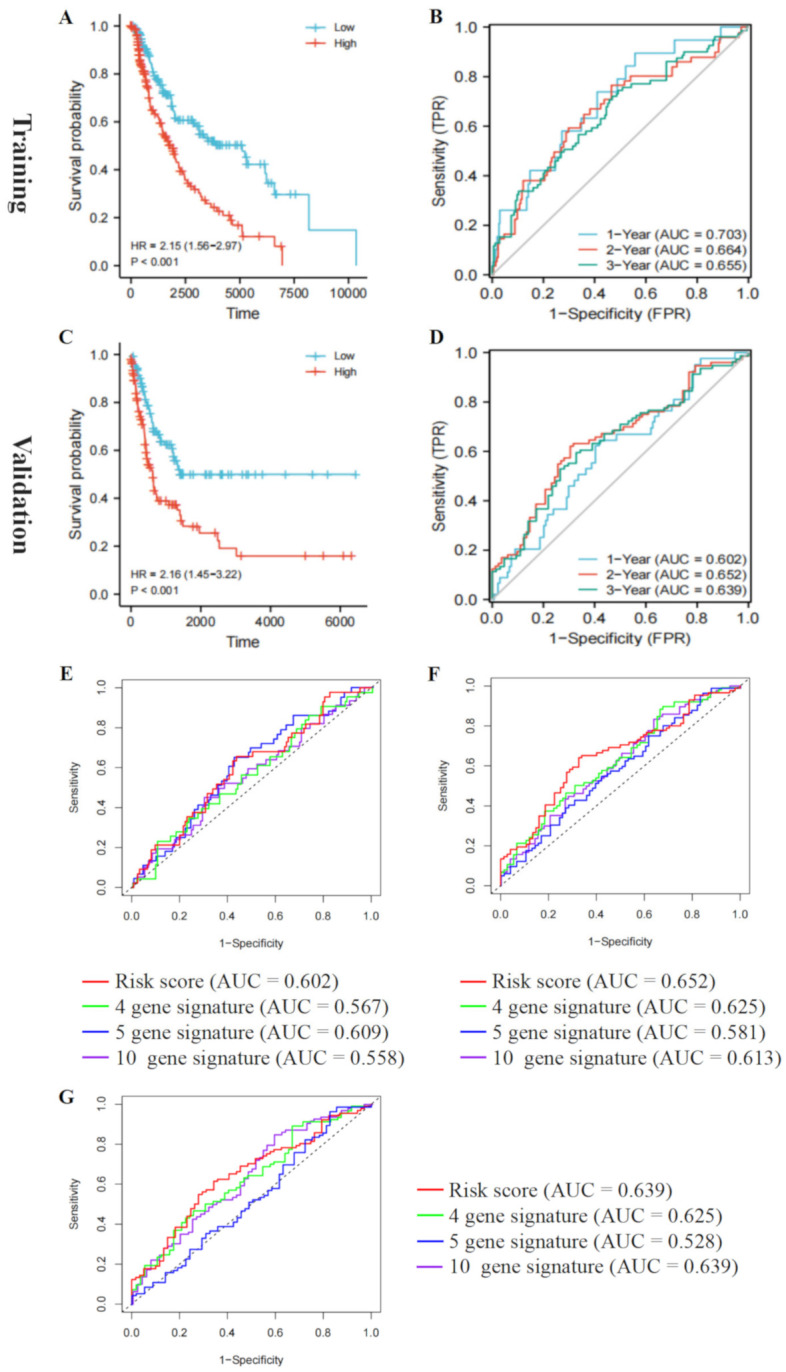
Validation of the prognostic risk signature. (**A**) In the training group, the OS of the two groups separated according to the median of risk scores of the prognostic risk signature was assessed. (**B**) The ROC curve was used to assess the prognostic feature predictive efficiency in the training group. (**C**) In the validation group, the OS of the two sets separated according to the median of risk scores of the prognostic risk signature was assessed. (**D**) The time-dependent ROC curve was used to evaluate the prediction efficiency of the prognostic model in the validation cohort. (**E**–**G**) The 1-year, 2-year, and 3-year AUC values of our RBM38-related immune signature were compared with the other three confirmed signatures of melanoma.

According to the median risk score, patients with malignant melanoma in the training and validation cohorts were assorted into low- and high-risk groups. The results revealed that patients with lower risk scores had better OS in the training and validation cohorts (*p* < 0.05; Figure 7A,C). Meanwhile, the time-dependent ROC curve showed that RAIPM had excellent prediction efficiency. The 1-, 2-, and 3-year AUC values for the training cohort were 0.703, 0.644, and 0.655, respectively (Figure 7B), and the 1-, 2-, and 3-year AUC values of ROC curves for the validation cohort were 0.602, 0.652, and 0.639, respectively (Figure 7D). As shown in Figure S5, low-risk patients had better clinical outcomes compared to high-risk patients (*p* < 0.05) in all subgroups (age ≤ 60, age > 60, female, male, stage I–II, and stage III–IV) in the training cohort. Subgroup analysis yielded consistent results showing that low-risk patients had better OS in all subgroups (age ≤ 60, age > 60, female, male, metastasis, and node) in the validation cohort (Figure S6). Altogether, these results reveal that RAIPM could identify malignant melanoma patients with higher risk scores associated with worse clinical outcomes.

The AUC values of risk score, age, gender, clinical stage, tumor, metastasis, and node in the training cohort were 0.662, 0.569, 0.489, 0.639, 0.674, 0.503, and 0.659, respectively (Figure S7A). The AUC values of risk score, gender, age, clinical stage, and tissue in the validation cohort were 0.639, 0.546, 0.470, 0.637, and 0.492, respectively (Figure S7B). This indicates that RAIPM had better melanoma prognostic ability than other clinical factors.

To compare the prognosis ability of RAIPM with the three other confirmed malignant melanoma prognostic signatures, ROC analyses of the other signatures were conducted similarly to the RAIPM analysis [26,27,28]. The AUC values of ROC analyses of RAIPM were 0.602, 0.652, and 0.639 for 1-, 2-, and 3- year OS, respectively. These were higher than the values for the 4-gene signature (1-, 2-, 3- year: AUC = 0.567, 0.625, 0.625), 5-gene signature (1-, 2- 3-year: AUC = 0.609, 0.581, 0.528), and 10-gene signature (1-, 2-, 3-year: AUC = 0.558, 0.613, 0.639) (Figure 7E–G). Therefore, our RAIPM risk score was a better predictor of prognosis in malignant melanoma.

### 3.7. Association between Immunosuppressive Molecules and Risk Scores in This Model

The immunohistochemical results of the Human Protein Atlas dataset demonstrated that four hub genes (A2M, NAMPT, LIF, and ERAP1) are overexpressed in melanoma tissues (Figure S7C). Significantly, the higher expressed pattern in melanoma tissues of the five genes was validated in our clinical samples (tumor vs. normal, *n* = 13). The results demonstrated that EBI3, ERAP1, and NAMPT mRNA levels were upregulated in melanoma tissues, while A2M and LIF were not insignificant (Figure S7D–H).

**Figure 8:**
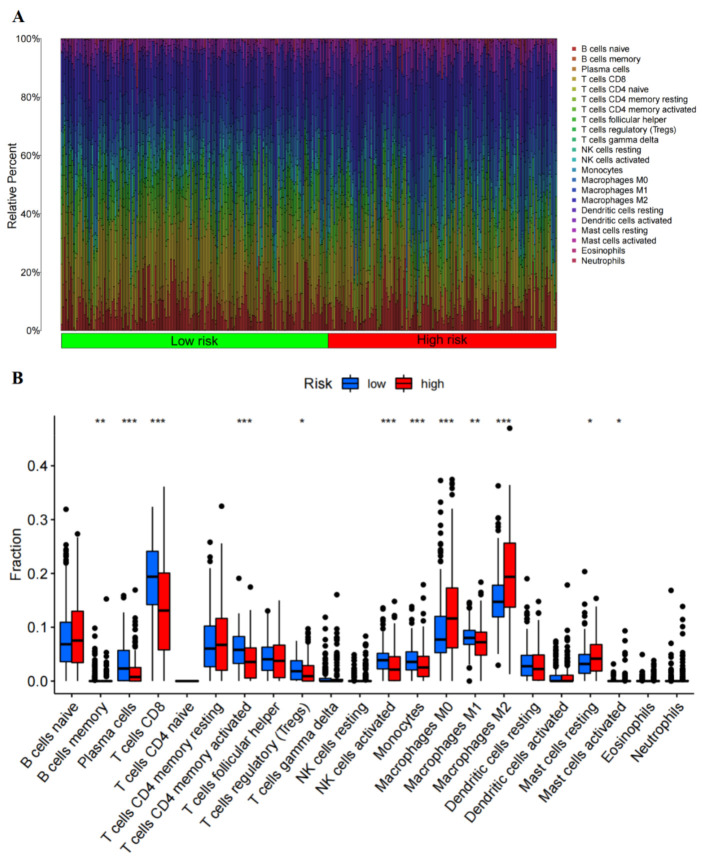
The immune infiltration of malignant melanoma patients with high and low-risk scores and the correlation between immune cell infiltration and risk score. (**A**) Bar plot demonstrating the relative proportion of 22 types of infiltrating immune cells in malignant melanoma patients with a high and low-risk score. (**B**) Box plot demonstrating the ratio differences of 22 types of immune cells in malignant melanoma patients with a high and low-risk score (* *p* < 0.05, ** *p* < 0.01, *** *p* < 0.001).

Characteristics of malignant melanoma patients related to the immune landscape, including the clinicopathological characteristics of RAIPM subgroups, are shown in Figure 8A. We found that macrophages M0 and M2 and resting mast cells were more abundant in the subgroup with higher risk, while plasma cells, activated memory CD4^+^ T cells, CD8^+^ T cells, Tregs, activated NK cells, monocytes, and M1 macrophages were more abundant in the subgroup with lower risk (Figure 8B).

**Figure 9:**
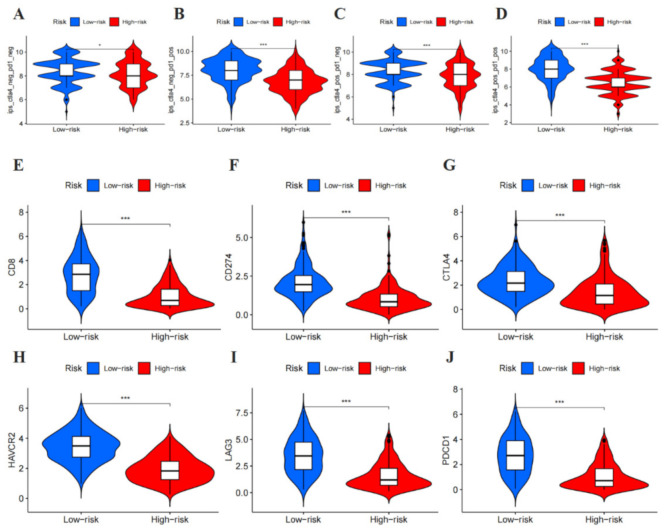
Immunosuppressed molecules and therapy associated with the signature-based risk score. (**A**–**D**) Immunophenoscore comparison between high-risk and low-risk groups in malignant melanoma patients in the PD-1 negative/positive or CTLA4 negative/positive groups. PD1 positive or CTLA4 positive represented anti-PD-1/PD-L1 or anti-CTLA4 therapy, respectively. (**E**) CD8, (**F**) CD274, (**G**) CTLA4, (**H**) HAVCR2, (**I**) LAG3, and (**J**) PDCD1 levels, the low-risk group was found to be positively correlated with upregulated, showed significant statistical difference in patients with melanoma. (* *p* < 0.05, *** *p* < 0.001).

The immunophenoscore (IPS) in the Cancer Immunome Atlas was used as a general index of immune activation in malignant melanoma samples. IPS is widely used to assess immunogenicity [29]. Melanoma patients in the low-risk group exhibited higher IPS (*p* < 0.05) (Figure 9A–D). Furthermore, the expression of ICIs between high- and low-risk melanoma patients were analyzed, indicating that the expression of immune-regulatory proteins (IRPs) such as PD-1 (PDCD1), PD-L1 (CD274), CTLA-4, HAVCR2 (TIM3), CD8, and LAG3 was significantly lower in the high-risk group (*p* < 0.05) (Figure 9E–J). Briefly, these results demonstrate that melanoma patients in the low-risk group appeared to have a higher positive response to immunotherapy and might receive more benefits.

## 4. Discussion

The development of malignant melanoma entails multiple activities that involve interactions among genetic, environmental, and host factors [30]. Malignant melanoma is also deemed one of the most immunogenic cancers and its relationship with immune cells in TME has a pivotal effect on the proliferation, progression, and metastasis of melanoma cells [31]. As it is an immunogenic tumor, immunotherapy for melanoma patients could be particularly attractive.

Recent studies revealed that aberrant expression of RBM38 was related to high malignancy and worse OS in various cancers, and its target genes were involved in regulating the process that results in malignant phenotypes such as tumor cell proliferation, invasion, and metastasis [32,33,34,35,36,37,38,39]. Our research found that melanoma patients with lower expression of RBM38 had a better prognosis in the TCGA-SKCM and GSE22155 datasets. Cox regression analysis was then used to assess RBM38 as an independent prognostic factor. It was subsequently confirmed that RBM38 plays a role in promoting the proliferation, invasion, and migration of malignant melanoma cells in vitro and the growth of human xenograft tumors in nude mice.

Consequently, the “amplification” alteration may be relevant to the function of RBM38 in melanoma. Moreover, the IHC analysis showed that the protein level of RBM38 was higher in human tumor tissues than in normal tissues. These results suggested that targeting RBM38 and its related pathways could provide probable strategies for molecular-based therapy. This study also indicated that B cell, neutrophils, and dendritic cells were concomitantly expressed with RBM38 through the analysis of the TIMER dataset and IHC staining.

From previously published studies, immune infiltration is known as a vital factor in TME, and immunotherapy significantly affects the prognosis. For example, it is reported that the prognosis of uveal melanoma patients is closely related to TME [40]. Meanwhile, studies also highlight that some prognostic signatures in melanoma were associated with immune infiltration [27,41,42]. Moreover, RBM38 was reported to form immune complexes with p21 or HuR mRNAs [10,15]. Consistent with these studies, our research revealed that immune molecular mechanisms were involved in the process of RBM38 promoting the proliferation and migration of melanoma cells.

Therefore, RNA sequencing assay and GO and KEGG analyses were utilized to analyze the RBM38-related DEGs and potential pathways. An RBM38-associated immune prognostic model (RAIPM) was constructed with the DEGs related to the immune pathway. In our research, the risk score of RAIPM could act as an independent prognostic biomarker and effective clinicopathological parameter predictor. Moreover, the external GSE65904 dataset was exerted to verify our model and the results make our model more convincing. RAIPM also has a better prognostic ability than other melanoma prognostic signatures. A recently published similar prognostic research started their article to analyze the whole RBP family genes [43]. Differently, our research was more focused on RBM38 and its association with prognostic models. Meanwhile, experiments in vivo and in vitro were performed to investigate the role of RBM38 in melanoma. Our results indicated that RBM38 might act as an oncogene in the progression of melanoma. Furthermore, the five critical genes of the prognostic model were verified to be more highly expressed in tumor tissue than normal tissues, not only in the Human Protein Atlas database. Moreover, the RT-PCR analysis in our clinical samples demonstrated that EBI3, ERAP1, and NAMPT mRNA expression levels were significantly upregulated in melanoma tissues compared to normal tissues.

Various immune cells in TME were involved in cancer prognosis, including adaptive immune CD8^+^/CD4^+^ T-lymphocytes, NK cells, MDSCs, dendritic cells, macrophages, and so on [44,45]. For instance, the link between human melanoma lesions and infiltration of T lymphocytes was associated with better OS [46]. Meanwhile, the infiltration of CD8^+^ T cells into tumor tissues was correlated with the effect of tumor treatment [47] and improved OS in some patients, most prominently in adjusting melanoma [48,49]. Immunoheterogeneity affects patients’ survival rate and immunotherapy outcome [50]. In line with these reports, the present research also revealed that many immune cells were involved in melanoma progression, including macrophages and CD8^+^ T lymphocytes. The risk score of RAIPM was also significantly connected to the infiltration level of various immune cells (macrophages, CD4^+^ and CD8^+^ T cells, etc.).

Typical immune checkpoint molecules such as CTLA-4 and PD-1/PD-L1 are related to the progress and prognosis of malignant melanoma [51,52,53]. PD-L1 expression in patients with melanoma has been reported to be more closely associated with clinical benefits from immunotherapy [54]. In our research, IPS and IRP (PD-1, LAG3, CTLA4, HAVCR4, and PD-L1) expression among patients were proved to be higher in the low-risk group, and greater efficacy of immune checkpoint block (ICB) treatment was observed in this group. These may demonstrate that immunotherapy is more suitable for malignant melanoma patients with low-risk RAIPM scores.

In conclusion, the five-gene signature could act as a prognostic biomarker and guide immunotherapy for melanoma and could even be utilized as a potential therapeutic target for melanoma in the future. The study is innovative in three aspects. To the best of our knowledge, our research is the first to identify the function of RBM38 in melanoma. Subsequently, the xenograft tumor in nude mice experiments confirmed that RBM38 promoted melanoma cell growth. In addition, IHC analysis confirmed the higher RBM38 expression in tumor tissues than normal tissues. Furthermore, RNA-seq analysis was used to determine the DEGs related to RBM38 and the RBM38-associated immune signature was established to predict melanoma risk.

Undeniably, there were some limitations to this research. Firstly, the immune cell infiltration in the animal study should be exerted to improve the results in future studies. Secondly, more melanoma cell lines could be conducted to investigate the role of RBM38 in melanoma in vivo and in vitro. Lastly, further preclinical trials are required to reveal the molecular mechanisms of the five hub genes in the progression of melanoma and to evaluate the predictive efficiency of the RAIPM risk score before clinical application.

## 5. Conclusions

For the first time, aberrant expression of RBM38 was found to be closely related to the malignant progression of melanoma in vivo and in vitro. An RBM38-associated immune gene model was established, and higher efficacy of ICB treatment was achieved in melanoma patients classified as low risk. This research may improve our understanding of the molecular mechanisms of melanoma occurrence and development. Furthermore, it could provide a unique perspective for discovering predictive biomarkers and searching for immunological and targeted therapies for melanoma.

## Figures and Tables

**Table 1:** The coefficients assessed by multivariate Cox regression.

Id	Coef	HR	HR.95L	HR.95H	*p*-Value
A2M	−0.097	0.908	0.824	1.000	0.050
NAMPT	−0.185	0.831	0.688	1.004	0.055
LIF	−0.123	0.885	0.809	0.967	0.007
EBI3	−0.247	0.781	0.674	0.905	0.001
ERAP1	−0.170	0.844	0.668	1.066	0.155

## Data Availability

All data generated or analyzed in this study are contained in this article or additional information files. The author will provide the raw data supporting the conclusions of this article to qualified researchers without reservation.

## Supplementary Materials

The following supporting information can be downloaded at: https://www.mdpi.com/article/10.3390/cancers14061590/s1. Table S1: RBM38 shRNA sequences. Table S2: Main reagents and consumables used in this article. Table S3: Clinical information of malignant melanoma patients in the TCGA-SKCM dataset. Table S4: Clinical information of malignant melanoma patients in the GSE65904 dataset. Table S5: Information of melanoma patients in the training group. Table S6: Information of melanoma patients in the validation group. Figure S1: Mutation feature of RBM38 in melanoma. We analyzed the mutation features of RBM38 for melanoma using the cBioPortal tool. (A) The alteration frequency with the mutation site is displayed. (B) The alteration frequency with mutation type is displayed. (C) The correlation between RBM38 and mutation types. (D) Overall survival analysis of the altered and unaltered group. Figure S2: RBM38 promoted the proliferation of malignant melanoma cells in vitro. (A–D) RT-qPCR analysis confirmed RBM38 overexpression and knockdown after transfection with lentivirus in A375 and M14 cell lines. (Data are shown as the mean ± SD of three replicates. * *p* < 0.05, ** *p* < 0.01, *** *p* < 0.001 by ANOVA test). (E–H) Effects of RBM38 knockdown and overexpression in M14 cells by wound healing assay on cell migration. (Data are expressed as the mean ± standard deviation of three replicates). (I) Representative images of RBM38, CD19, CD11b, and E-cadherin staining in melanoma tissues (*n* = 17) by IHC staining. Scale bars indicated 25 μm. In addition, RBM38 is correlated with CD19, CD11b, and E-cadherin. (J) The pheatmap of the correlation of RBM38, CD11b, CD19, and E-cadherin of Immunohistochemical staining analysis (* *p* < 0.05, ** *p* < 0.01, *** *p* < 0.001), in addition, RBM38 is correlated with CD19, CD11b, and E-cadherin. (K) The correlation between RBM38 and E-cadherin of Immunohistochemical staining analysis. (L) The correlation between RBM38 and CD11b of Immunohistochemical staining analysis. (M) The correlation between RBM38 and CD19 of Immunohistochemical staining analysis. (For statistical comparison between two independent experimental groups (Student’s *t*-test) and among more than two experimental groups (ANOVA test), appropriated statistical tests were assayed * *p* < 0.05, ** *p* < 0.01, *** *p* < 0.001). Figure S3: Representative images of RBM38, CD19, CD11b, and E-cadherin staining. (A) Other 17 paired images of RBM38 staining in melanoma and normal tissues. (B) Representative images of the negative control of the tumor and normal tissue staining. Figure S4: The relevance of immune cells in the TME and the correlation of TiL/TiM, ICB, and RBM38. (A) The immune cells exhibited intricate relevance among each other in the TME (* *p* < 0.05, ** *p* < 0.01, *** *p* < 0.001). (B) The results showed that RBM38 is correlated with ICB and not with TiL and TiM through PCA analysis. (C) A total of 158 downregulated genes were differentially identified from mRNA sequencing. Figure S5: The OS of the low- and high-risk subgroups separated by clinicopathological parameters in the training group. (A,B) In the training group, the OS of the low- and high-risk subgroups separated by age were assessed. (C,D) In the training group, the OS of the low- and high-risk subgroups separated by gender were assessed. (E,F) In the training group, the OS of the low- and high-risk subgroups separated by stage were assessed. Figure S6: The OS of the low- and high-risk subgroups separated by clinicopathological parameters in the validation group. (A,B) In the validation group, the OS of the low- and high-risk subgroups separated by age were assessed. (C,D) In the validation group, the OS of the low- and high-risk subgroups separated by gender were assessed. (E,F) In the validation group, the OS of the low- and high-risk subgroups separated by metastasis and node were assessed. Figure S7: Validation of the five-gene signature and immunohistochemical assay. (A,B) In the training and validation cohort, risk score, age, gender, clinical stage, tumor, metastasis, and AUC value of nodes with clinical information were explored. (C) Protein expression of five hub genes (A2M, NAMPT, LIF, EBI3, and ERAP1) was assessed by an immunohistochemical assay in malignant melanoma, according to the Human Protein Atlas website. (D–H) RT-qPCR analysis of A2M, EBI3, NAMPT, LIF, and ERAP1 expressions of mRNA in 13 paired melanoma tissues and matched normal tissues quantified after transfection. (Data are shown as the mean ± SD of three replicates. * *p* < 0.05, ** *p* < 0.01, *** *p* < 0.001 by ANOVA test).

## Abbreviations

RBP, RNA binding proteins; RBM38, RNA-binding protein 1; A2M, Alpha-2-macroglobulin; EBI3, Epstein-Barr virus-induced gene3; ERAP1, Endoplasmic reticulum aminopeptidase1; DEGs, differentially expressed genes; FDR, false discovery rate; IHC, Immunohistochemical; IPS, immunophenoscore; ICIs, immune checkpoint inhibitors; ICB, immune checkpoint blockade; LIF, leukemia inhibitory factor; NAMPT, human skin fibroblasts; RRM, RNA recognition motif; RAIPM, RBM38-associated immune prognostic model; OS, overall survival; SKCM, skin cutaneous melanoma; TIMER, Tumor Immune Estimation Resource; TME, tumor microenvironment.
