# Supplementary material for: Immune Landscape and an RBM38-Associated Immune Prognostic Model with Laboratory Verification in Malignant Melanoma
Source: Cancers (Basel). 2022 Mar 21;14(6):1590. doi: 10.3390/cancers14061590 (PMC8946480; doi:10.3390/cancers14061590)
Supplement: Supplementary file 1 [file cancers-14-01590-s001.zip › cancers-1640999-supplementary.pdf]

cancers

MDPI

Supplementary Material

Immune Landscape and an RBM38-Associated Immune Prognostic Model with Laboratory Verification in Malignant Melanoma

Jinfang Liu, Jun Xu, Binlin Luo, Jian Tang, Zuoqiong Hou, Zhechen Zhu, Lingjun Zhu, Gang Yao and Chujun Li

1. Supplementary Tables

Table S1. NBM38 shRNA sequences.

Group	5'-3' sequence
Sh1	CGCCATGCACGGCTCGCAGAAGGACACCA
Sh2	GGCACCACTTTCGTGCAGTACCAGGCGCC

Table S2. Main reagents and consumable used in this article.

Reagents	Vendor	Catalogue Number	Information
Antibody-RBM38	BIOSS	bs-7682R	Huaman, Polyclonal, IHC (1:100)
Antibody-CD11b	ProteinTech	66519-1-lg	Human, Monoclonal, IHC (1:100)
Antibody-CD19	ProteinTech	66298-1-lg	Huaman, Monoclonal, IHC (1:100)
Antibody-E-cadherin	ProteinTech	60335-1-lg	Huaman, Monoclonal, IHC (1:100)
Primer-Actin	Tsingke Biotechnology	/	Forward: 5′-AGAAAATCTGGCACCACACC-3′ Reverse: 5′-TAGCACAGCCTGGATAGCAA-3′
Primer-RBM38	Tsingke Biotechnology	/	Forward: 5′- ACGCCTCGCTCAGGAAGTA -3′ Reverse: 5′- GTCTTTGCAAGCCCTCTCAG -3
Primer-A2M	Tsingke Biotechnology	/	Forward: 5′- CGGAGAATGACGTACTCCACT -3′ Reverse: 5′- TGGGTTGGTCCTTTCACTTGG -3
Primer-EBI3	Tsingke Biotechnology	/	Forward: 5′- TCATTGCCACGTACAGGCTC -3′ Reverse: 5′- GGGTCGGGCTTGATGATGTG -3
Primer-ERAP1	Tsingke Biotechnology	/	Forward: 5′- AGAGCACTGAAGCATCTCCAA -3′ Reverse: 5′- AACTGGGATGACGTACTCAGG -3
Primer-NAMPT	Tsingke Biotechnology	/	Forward: 5′- ATCCTGTTCCAGGCTATTCTGT -3′ Reverse: 5′- CCCCATATTTTCTCACACGCAT -3

Primer-LIF	Tsingke Biotechnology	/	Forward:<br>5′- CCAACGTGACGGACTTCCC -3′<br>Reverse:<br>5′- TACACGACTATGCGGTACAGC -3

Abbreviations: RBM38: RNA-binding protein 1; A2M: Alpha-2-macroglobulin; EBI3: Epstein-Barr virus-induced gene3; ERAP1: Endoplasmic reticulum aminopeptidase1; NAMPT: human skin fibroblasts; LIF: leukemia inhibitory factor.

Table S3. Clinical information of malignant melanoma patients in TCGA-SKCM dataset.

Variables	Group	Alive (n = 252)	Dead (n = 208)	Total (n = 460)
Age	≤60	134 (53.2%)	115 (55.3%)	249 (54.1%)
	>60	118 (46.8%)	93 (44.7%)	211 (45.9%)
Gender	Female	107 (42.5%)	68 (32.7%)	175 (38.0%)
	Male	145 (57.5%)	140 (67.3%)	285 (62.0%)
Stage	<stage III	130 (51.6%)	102 (49.0%)	232 (50.4%)
	≥stage III	122 (48.4%)	106 (51.0%)	228 (49.6%)
Clinical_T	unknow	12 (4.8%)	15 (7.2%)	27 (5.9%)
	TX	24 (9.5%)	20 (9.6%)	44 (9.6%)
	<T3	84 (33.3%)	64 (30.8%)	148 (32.2%)
	≥T3	132 (52.4%)	109 (52.4%)	241 (52.4%)
Clinical_N	unknow	10 (4.0%)	9 (4.3%)	19 (4.1%)
	NX	21 (8.3%)	14 (6.7%)	35 (7.6%)
	N0	124 (49.2%)	105 (50.5%)	229 (49.8%)
	≥N1	97 (38.5%)	80 (38.5%)	177 (38.5%)
Clinical_M	unknow	15 (6.0%)	11 (5.3%)	26 (5.7%)
	M0	223 (88.5%)	188 (90.4%)	411 (89.3%)
	M1	14 (5.6%)	9 (4.3%)	23 (5.0%)

Abbreviations: Clinical_T, Clinical Tumor; TX, T stage unknown; T3, Tumor thickness: 2.01 mm-4.0 mm; NX, T stage unknown; N0, metastatic lymph node presents; N1, 1 metastatic lymph node presents, lymph node size <=3cm; M0, without distant metastasis; M1, with distant metastasis.

Table S4. Clinical information of malignant melanoma patients in GSE65904 dataset.

Variables	Group	Alive (n = 108)	Dead (n = 102)	Total (n = 210)
Age	unknow	1 (0.9%)	0 (0%)	1 (0.5%)
	≤60	40 (37.0%)	43 (42.2%)	83 (39.5%)
	>60	67 (62.0%)	59 (57.8%)	126 (60.0%)
Gender	Female	52 (48.1%)	34 (33.3%)	86 (41.0%)
	Male	56 (51.9%)	68 (66.7%)	124 (59.0%)
Tumor Site	unknow	4 (3.7%)	3 (2.9%)	7 (3.3%)
	General	7 (6.5%)	16 (15.7%)	23 (11.0%)
	In-transit	5 (4.6%)	10 (9.8%)	15 (7.1%)
	Local	7 (6.5%)	4 (3.9%)	11 (5.2%)
	Primary	14 (13.0%)	1 (1.0%)	15 (7.1%)
	Regional	71 (65.7%)	68 (66.7%)	139 (66.2%)
Tissue	unknow	6 (5.6%)	9 (8.8%)	15 (7.1%)
	Cutaneous	18 (16.7%)	3 (2.9%)	21 (10.0%)
	Lymph node	67 (62.0%)	63 (61.8%)	130 (61.9%)
	Subcutaneous	13 (12.0%)	20 (19.6%)	33 (15.7%)
	Visceral	4 (3.7%)	6 (5.9%)	10 (4.8%)
	Other	0 (0%)	1 (1.0%)	1 (0.5%)

Table S5. Information of melanoma patients in the training group.

Id	Futime	Fustat
TCGA-D3-A1Q1-06A	1.380821918	1
TCGA-EB-A5SH-06A	4.501369863	0
TCGA-FS-A1Z0-06A	16.88767123	1
TCGA-EB-A4P0-01A	0.893150685	1
TCGA-FR-A3YN-06A	7.747945205	0
TCGA-FS-A4FD-06A	6.723287671	1
TCGA-DA-A95Y-06A	1.178082192	1
TCGA-EE-A3J8-06A	2.860273973	1
TCGA-FS-A1ZG-06A	0.808219178	1
TCGA-GN-A268-06A	5.232876712	1
TCGA-EE-A2GC-06A	5.619178082	0
TCGA-EE-A2GT-06A	3.739726027	0
TCGA-EE-A2GJ-06A	6.219178082	0
TCGA-EE-A2M6-06A	10.77260274	0
TCGA-ER-A2NC-06A	3.652054795	1
TCGA-D3-A2JA-06A	9.62739726	0
TCGA-DA-A1HW-06A	1.17260274	0
TCGA-FS-A1ZJ-06A	3.947945205	1
TCGA-EE-A20H-06A	14.02191781	1
TCGA-GN-A4U9-06A	1.843835616	1
TCGA-D9-A4Z3-01A	1.383561644	0
TCGA-EE-A2MS-06A	13.53972603	0
TCGA-FS-A1ZD-06A	4.460273973	1
TCGA-DA-A1IC-06A	5.673972603	1
TCGA-EE-A17X-06A	2.484931507	1
TCGA-ER-A19D-06A	1.049315068	1
TCGA-D3-A1Q3-06A	1.389041096	1

TCGA-D9-A6EG-06A	1.912328767	1
TCGA-EE-A2MF-06A	22.39452055	1
TCGA-EE-A3J4-06A	10.6	1
TCGA-EE-A2MR-06A	11.2	0
TCGA-EE-A2GM-06B	6.290410959	0
TCGA-EE-A3JI-06A	12.73424658	1
TCGA-FR-A8YC-06A	2.901369863	1
TCGA-D3-A8GP-06A	12.70684932	0
TCGA-YG-AA3P-06A	1.202739726	0
TCGA-WE-A8ZT-06A	0.983561644	0
TCGA-GN-A26C-01A	2.249315068	1
TCGA-D3-A51K-06A	2.745205479	0
TCGA-EE-A2GS-06A	6.767123288	1
TCGA-D3-A3MU-06A	3.312328767	0
TCGA-FS-A4FB-06A	2.22739726	1
TCGA-DA-A1I8-06A	3.747945205	0
TCGA-D3-A5GT-01A	1.334246575	0
TCGA-EE-A3J7-06A	5.339726027	0
TCGA-D9-A4Z6-06A	1.536986301	1
TCGA-EE-A2GB-06A	4.939726027	0
TCGA-FS-A4F0-06A	6.484931507	0
TCGA-EE-A3AC-06A	4.301369863	0
TCGA-FS-A1ZW-06A	4.123287671	0
TCGA-EB-A42Z-01A	1.208219178	0
TCGA-Z2-AA3S-06A	8.082191781	0
TCGA-D3-A3MV-06A	3.775342466	0
TCGA-D3-A1Q6-06A	5.983561644	1
TCGA-EE-A2GR-06A	3.564383562	1
TCGA-WE-A8K5-06A	5.095890411	1
TCGA-FR-A8YE-06A	8.701369863	0
TCGA-D3-A2J6-06A	3.619178082	1
TCGA-D3-A51R-06A	5.317808219	0
TCGA-EE-A2GE-06A	13.41917808	0
TCGA-DA-A3F8-06A	1.347945205	0
TCGA-D3-A8GS-06A	9.764383562	1
TCGA-EB-A5KH-06A	1.695890411	1
TCGA-D3-A3CF-06A	2.043835616	1
TCGA-EE-A3JA-06A	4.432876712	1
TCGA-EE-A2MK-06A	13.21917808	0
TCGA-FS-A1Z7-06A	0.649315068	1
TCGA-ER-A19T-01A	0.739726027	1
TCGA-FW-A3TV-06A	1.126027397	0
TCGA-EE-A29S-06A	5.106849315	1
TCGA-EE-A2M7-06A	2.402739726	1
TCGA-WE-A8ZR-06A	0.750684932	1
TCGA-BF-A5EP-01A	0.917808219	0
TCGA-EE-A2GI-06A	4.060273973	0
TCGA-D3-A5GS-06A	1.515068493	0
TCGA-Z2-AA3V-06A	1.331506849	0
TCGA-EE-A2MI-06A	17.05479452	1
TCGA-WE-A8ZO-06A	5.635616438	0

TCGA-D3-A51E-06A	14.56986301	0
TCGA-EE-A2ME-06A	8.605479452	1
TCGA-EE-A181-06A	2.810958904	1
TCGA-D3-A2JG-06A	9.460273973	1
TCGA-EE-A2GU-06A	7.901369863	0
TCGA-D3-A2J9-06A	1.980821918	1
TCGA-FR-A728-01A	1.597260274	0
TCGA-D3-A3ML-06A	1.156164384	1
TCGA-EB-A6R0-01A	1.279452055	0
TCGA-D3-A8GR-06A	10.80273973	1
TCGA-DA-A1I2-06A	13.93972603	0
TCGA-FS-A1ZY-06A	2.257534247	1
TCGA-EE-A3AA-06A	10.35890411	0
TCGA-EE-A2GN-06A	8.509589041	1
TCGA-EE-A184-06A	5.679452055	1
TCGA-FS-A4F4-06A	5.556164384	1
TCGA-EB-A551-01A	1.616438356	0
TCGA-WE-A8ZY-06A	1.01369863	0
TCGA-DA-A3F3-06A	0.873972603	1
TCGA-EB-A44Q-06A	1.134246575	0
TCGA-GN-A4U7-06A	0.868493151	1
TCGA-D3-A1QB-06A	7.978082192	0
TCGA-WE-A8ZQ-06A	4.980821918	0
TCGA-D3-A8GM-06A	8.928767123	1
TCGA-ER-A2ND-06A	1.945205479	1
TCGA-EE-A29M-06A	4.736986301	0
TCGA-WE-AAA0-06A	2.917808219	0
TCGA-EE-A2MN-06A	3.961643836	1
TCGA-FS-A4F8-06A	14.56986301	1
TCGA-3N-A9WD-06A	1.082191781	1
TCGA-HR-A2OH-06A	5.490410959	1
TCGA-GF-A3OT-06A	0.824657534	0
TCGA-FS-A1ZF-06A	1.287671233	1
TCGA-EE-A2MT-06A	5.934246575	0
TCGA-ER-A19E-06A	1.084931507	1
TCGA-FS-A1ZU-06A	2.21369863	1
TCGA-EE-A2GK-06A	4.561643836	0
TCGA-D9-A6E9-06A	0.824657534	0
TCGA-DA-A3F2-06A	1.8	0
TCGA-EE-A29H-06A	5.38630137	0
TCGA-EE-A29L-06A	0.216438356	1
TCGA-W3-A824-06A	19.01369863	0
TCGA-DA-A1I5-06A	8.98630137	0
TCGA-EE-A2GL-06A	6.638356164	0
TCGA-EB-A42Y-01A	1.205479452	0
TCGA-EE-A17Z-06A	0.720547945	1
TCGA-EE-A29E-06A	5.315068493	0
TCGA-EB-A41B-01A	0.797260274	0
TCGA-EB-A431-01A	1.556164384	0
TCGA-D3-A2J7-06A	8.591780822	1
TCGA-EE-A2A0-06A	3.901369863	1

TCGA-EE-A2MU-06A	4.438356164	0
TCGA-D9-A4Z5-01A	0.597260274	0
TCGA-DA-A1HY-06A	9.808219178	0
TCGA-EE-A2MQ-06A	3.602739726	1
TCGA-EE-A2MH-06A	1.41369863	1
TCGA-EE-A182-06A	1.224657534	1
TCGA-DA-A1I0-06A	1.62739726	0
TCGA-EE-A3AD-06A	2.397260274	1
TCGA-FS-A1Z4-06A	2.339726027	1
TCGA-DA-A1IB-06A	1.243835616	0
TCGA-DA-A960-01A	2.202739726	0
TCGA-EB-A24D-01A	1.767123288	0
TCGA-D3-A3MR-06A	8.632876712	0
TCGA-D3-A8GK-06A	14.18356164	0
TCGA-EE-A29D-06A	1.164383562	1
TCGA-EE-A2MD-06A	3.939726027	1
TCGA-BF-AAP2-01A	1.109589041	0
TCGA-EE-A17Y-06A	2.268493151	1
TCGA-D9-A3Z3-06A	1.857534247	0
TCGA-FS-A1ZZ-06A	2.252054795	1
TCGA-W3-AA1R-06A	9.257534247	1
TCGA-W3-AA1W-06A	18.2630137	0
TCGA-EE-A2A6-06A	7.178082192	0
TCGA-GN-A4U3-06A	10.15890411	0
TCGA-EE-A2MM-06A	13.99178082	1
TCGA-ER-A197-06A	1.161643836	1
TCGA-EE-A2M8-06A	1.646575342	1
TCGA-FS-A1YW-06A	18.07671233	1
TCGA-BF-A3DM-01A	1.646575342	0
TCGA-D3-A5GL-06A	10.48219178	0
TCGA-W3-AA21-06A	8.753424658	1
TCGA-EE-A3AH-06A	11.56712329	1
TCGA-EB-A4OY-01A	1.624657534	0
TCGA-ER-A2NE-06A	1.679452055	1
TCGA-EE-A2MG-06A	8.6	1
TCGA-DA-A1I1-06A	16.27671233	0
TCGA-ER-A3EV-06A	3.915068493	1
TCGA-D3-A2J8-06A	5.457534247	1
TCGA-D3-A2JB-06A	14	1
TCGA-Z2-A8RT-06A	2.298630137	0
TCGA-EB-A5VV-06A	0.58630137	0
TCGA-FR-A7UA-06A	3.189041096	0
TCGA-EE-A185-06A	0.41369863	1
TCGA-EE-A2ML-06A	18.05479452	1
TCGA-BF-A1PX-01A	0.77260274	1
TCGA-FR-A3R1-01A	1.876712329	0
TCGA-D3-A1QA-06A	7.575342466	0
TCGA-DA-A3F5-06A	17.66027397	0
TCGA-GF-A2C7-01A	0.057534247	0
TCGA-EE-A29V-06A	2.156164384	1
TCGA-EE-A2A2-06A	3.068493151	0

TCGA-EB-A97M-01A	1.134246575	0
TCGA-ER-A2NG-06A	4.082191781	1
TCGA-EE-A29X-06A	1.493150685	1
TCGA-FS-A1ZM-06A	8.438356164	0
TCGA-D3-A3CC-06A	7.243835616	0
TCGA-FS-A1ZK-06A	1.994520548	1
TCGA-ER-A3ET-06A	7.750684932	1
TCGA-EB-A44P-01A	1.030136986	0
TCGA-DA-A1HV-06A	4.115068493	0
TCGA-FR-A729-06A	18.4	0
TCGA-BF-A5ES-01A	1.342465753	0
TCGA-EE-A2GO-06A	10.56712329	0
TCGA-EB-A6QY-01A	1.046575342	0
TCGA-WE-A8JZ-06A	2.002739726	0
TCGA-BF-A1Q0-01A	2.276712329	0
TCGA-FS-A1YX-06A	4.049315068	1
TCGA-EE-A180-06A	7.915068493	1
TCGA-QB-A6FS-06A	0.602739726	0
TCGA-D3-A2JN-06A	5.539726027	1
TCGA-D3-A1Q8-06A	2.339726027	1
TCGA-EE-A2GH-06A	17.3369863	0
TCGA-D3-A51F-06A	4.643835616	0
TCGA-ER-A199-06A	0.764383562	1
TCGA-D9-A6EA-06A	2.098630137	0
TCGA-EB-A44O-01A	0.221917808	0
TCGA-W3-A828-06A	10.09041096	1
TCGA-EE-A29A-06A	5.279452055	1
TCGA-EE-A2M5-06A	1.805479452	1
TCGA-EE-A29B-06A	7.090410959	1
TCGA-EE-A3J5-06A	3.079452055	1
TCGA-EE-A3JB-06A	16.81643836	0
TCGA-D3-A5GR-06A	14.86027397	0
TCGA-BF-A5EQ-01A	0.884931507	0
TCGA-D9-A6EC-06A	5.520547945	0
TCGA-EE-A20C-06A	12.60547945	1
TCGA-FS-A4F9-06A	2.835616438	0
TCGA-FS-A1ZT-06A	4.430136986	0
TCGA-FS-A4F2-06A	4.178082192	1
TCGA-YG-AA3N-01A	0.838356164	0
TCGA-EB-A5VU-01A	0.879452055	1
TCGA-EE-A3AF-06A	1.150684932	1
TCGA-BF-AAP1-01A	1.120547945	0
TCGA-EB-A3Y6-01A	0.345205479	0
TCGA-FS-A4F5-06A	2.394520548	1
TCGA-D3-A1Q9-06A	2.632876712	1
TCGA-D3-A51T-06A	2.24109589	0
TCGA-EE-A2A5-06A	3.273972603	1
TCGA-FS-A1ZH-06A	2.728767123	1
TCGA-D3-A2JP-06A	4.964383562	0
TCGA-BF-A5ER-01A	0.895890411	0
TCGA-GF-A4EO-06A	1.619178082	0

TCGA-EE-A3JH-06A	9.131506849	0
TCGA-EE-A29R-06A	1.205479452	0
TCGA-ER-A19K-01A	1.284931507	1
TCGA-EB-A1NK-01A	1.679452055	0
TCGA-ER-A3PL-06A	2.767123288	0
TCGA-EE-A29P-06A	4.701369863	0
TCGA-D3-A8GL-06A	7.42739726	1
TCGA-D3-A3C6-06A	4.838356164	1
TCGA-EB-A82C-01A	0.046575342	0
TCGA-D9-A3Z1-06A	1.282191781	1
TCGA-FS-A1YY-06A	19.04931507	1
TCGA-D3-A2JH-06A	3.506849315	0
TCGA-BF-A1PZ-01A	2.336986301	0
TCGA-FR-A8YD-06A	3.021917808	1
TCGA-FS-A1ZA-06A	2.309589041	1
TCGA-EE-A183-06A	2.24109589	1
TCGA-D9-A4Z2-01A	0.520547945	1
TCGA-EB-A44N-01A	0.561643836	1
TCGA-EB-A3Y7-01A	0.893150685	1
TCGA-EE-A29W-06A	16.25205479	0
TCGA-D3-A51J-06A	12.09315068	0
TCGA-D3-A51H-06A	4.695890411	0
TCGA-FS-A1ZP-06A	6.22739726	1
TCGA-EE-A2GP-06A	1.15890411	1
TCGA-EE-A20F-06A	7.630136986	0
TCGA-FR-A44A-06A	14.51780822	0
TCGA-BF-A3DL-01A	2.106849315	0
TCGA-BF-A9VF-01A	1.205479452	0
TCGA-D3-A8GJ-06A	20.11506849	0
TCGA-BF-AAOX-01A	1.216438356	0
TCGA-EE-A3J3-06A	14.34794521	1
TCGA-WE-A8K1-06A	4.087671233	0
TCGA-XV-AAZW-01A	1.076712329	1
TCGA-D3-A3CB-06A	13.87671233	0
TCGA-BF-A1PU-01A	1.060273973	0
TCGA-EB-A3XE-01A	0.493150685	0
TCGA-D3-A1Q7-06A	11.10410959	0
TCGA-BF-AAP6-01A	0.890410959	0
TCGA-EE-A29Q-06A	5.561643836	1
TCGA-GN-A263-01A	1.279452055	1
TCGA-D3-A8GI-06A	4.876712329	1
TCGA-XV-AAZV-01A	1.128767123	0
TCGA-FS-A1ZS-06A	12.4	0
TCGA-D3-A3BZ-06A	10.89315068	0
TCGA-ER-A2NH-06A	3.463013699	0
TCGA-EB-A5UM-01A	1.134246575	0
TCGA-FR-A2OS-01A	1.008219178	1
TCGA-BF-A3DN-01A	1.964383562	0
TCGA-D3-A5GN-06A	11.31232877	0
TCGA-GN-A26A-06A	2.706849315	1
TCGA-EB-A6QZ-01A	0.964383562	1

TCGA-D9-A3Z4-01A	1.421917808	1
TCGA-DA-A1I4-06A	2.994520548	1
TCGA-EB-A85J-01A	0.98630137	0
TCGA-WE-AAA3-06A	1.643835616	0
TCGA-EB-A3XF-01A	0.761643836	0
TCGA-D3-A5GU-06A	10.43287671	0
TCGA-D3-A8GD-06A	1.967123288	0
TCGA-FR-A7U9-06A	1.564383562	0
TCGA-DA-A1I7-06A	5.139726027	0
TCGA-EE-A2GD-06A	28.34520548	1
TCGA-W3-A825-06A	5.252054795	1
TCGA-ER-A2NF-01A	2.402739726	1
TCGA-DA-A1IA-06A	5.493150685	1
TCGA-BF-AAP4-01A	0.917808219	0
TCGA-DA-A95X-06A	4.145205479	0
TCGA-FS-A1ZN-01A	2	1
TCGA-EB-A85I-01A	0.991780822	0
TCGA-GN-A267-06A	5.369863014	1
TCGA-D3-A2JC-06A	7.230136986	0
TCGA-ER-A19M-06A	5.087671233	1
TCGA-ER-A42K-06A	1.079452055	1
TCGA-W3-AA1V-06B	3.506849315	1
TCGA-FS-A1ZB-06A	4.071232877	1
TCGA-EB-A4OZ-01A	1.698630137	0
TCGA-EB-A3XC-01A	1.780821918	0
TCGA-FS-A1ZE-06A	3.871232877	1
TCGA-D3-A3CE-06A	5.019178082	1
TCGA-EE-A2A1-06A	8.575342466	0
TCGA-D3-A1Q4-06A	9.336986301	0
TCGA-XV-A9W5-01A	1.073972603	0
TCGA-FS-A1ZR-06A	0.950684932	1
TCGA-EE-A2MC-06A	5.126027397	1
TCGA-EB-A3HV-01A	0.106849315	0
TCGA-FS-A4FC-06A	4.534246575	1
TCGA-BF-A5EO-01A	1.926027397	0
TCGA-IH-A3EA-01A	1.435616438	0
TCGA-ER-A2NB-01A	2.347945205	1
TCGA-EE-A3JE-06A	4.279452055	0
TCGA-ER-A193-06A	2.616438356	1
TCGA-D3-A2JD-06A	0.989041096	1
TCGA-ER-A196-01A	4.890410959	0
TCGA-EB-A82B-01A	1.068493151	0
TCGA-BF-A1PV-01A	0.038356164	0
TCGA-EE-A20B-06A	11.15068493	0
TCGA-XV-A9W2-01A	1.142465753	0
TCGA-D3-A2JF-06A	5.17260274	0
TCGA-EE-A2MP-06A	20.72054795	0
TCGA-D3-A51N-06A	1.884931507	0
TCGA-EE-A29C-06A	6.580821918	1
TCGA-BF-AAP7-01A	0.871232877	0
TCGA-EE-A2MJ-06A	8.019178082	1

TCGA-XV-AAZY-01A	1.109589041	0
TCGA-D3-A2JK-06A	1.008219178	1
TCGA-ER-A42L-06A	12.41917808	0
TCGA-D3-A8GB-06A	2.569863014	1
TCGA-BF-A3DJ-01A	1.271232877	0
TCGA-D3-A8GQ-06A	2.421917808	1
TCGA-BF-AAP8-01A	1.224657534	0
TCGA-D3-A3C7-06A	3.915068493	0
TCGA-D3-A5GO-06A	11.49315068	0
TCGA-EB-A553-01A	0.619178082	0
TCGA-EE-A3AG-06A	3.465753425	1
TCGA-EB-A57M-01A	1.093150685	0
TCGA-EB-A299-01A	1.035616438	0
TCGA-BF-AAOU-01A	1.304109589	0
TCGA-EE-A29G-06A	6.005479452	1
TCGA-EE-A3AB-06A	10.22739726	0

Table S6. Information of melanoma patients in the validation group.

Id	Futime	Fustat
GSM1608593	4.695890411	0
GSM1608594	0.967123288	1
GSM1608595	5.82739726	0
GSM1608596	3.950684932	0
GSM1608597	0.180821918	1
GSM1608598	7.208219178	0
GSM1608599	6.649315068	0
GSM1608600	3.589041096	0
GSM1608601	2.238356164	0
GSM1608602	3.838356164	1
GSM1608603	0.276712329	0
GSM1608604	8.276712329	1
GSM1608605	1.761643836	1
GSM1608606	3.682191781	1
GSM1608607	3.016438356	0
GSM1608608	0.205479452	0
GSM1608609	0.210958904	1
GSM1608610	4.068493151	1
GSM1608611	0.254794521	0
GSM1608612	2.326027397	1
GSM1608613	0.468493151	0
GSM1608614	0.534246575	1
GSM1608615	3.468493151	0
GSM1608616	17.37808219	0
GSM1608617	2.246575342	0
GSM1608619	0.517808219	0
GSM1608620	1.24109589	1
GSM1608621	3.342465753	0
GSM1608622	1.531506849	0
GSM1608623	2.284931507	1
GSM1608624	2.821917808	0
GSM1608625	8.783561644	0

GSM1608626	3.284931507	1
GSM1608627	14.27945205	0
GSM1608628	1.621917808	1
GSM1608629	0.136986301	0
GSM1608630	3.252054795	0
GSM1608631	1.147945205	1
GSM1608632	0.569863014	1
GSM1608633	6.797260274	1
GSM1608634	0.731506849	0
GSM1608635	1.191780822	1
GSM1608636	2.032876712	1
GSM1608637	0.265753425	1
GSM1608638	1.235616438	0
GSM1608639	10.35342466	0
GSM1608640	0.531506849	1
GSM1608641	7.052054795	0
GSM1608642	3.452054795	0
GSM1608643	1.736986301	0
GSM1608644	0.060273973	0
GSM1608645	1.619178082	1
GSM1608646	0.287671233	0
GSM1608647	3.98630137	0
GSM1608648	3.846575342	1
GSM1608649	1.035616438	1
GSM1608650	6.931506849	1
GSM1608651	1.506849315	1
GSM1608652	8.726027397	0
GSM1608653	0.849315068	1
GSM1608654	0.77260274	0
GSM1608655	9.082191781	0
GSM1608656	0.753424658	1
GSM1608657	9.81369863	0
GSM1608658	1.326027397	0
GSM1608659	2.002739726	0
GSM1608660	2.397260274	0
GSM1608661	0.032876712	1
GSM1608662	1.75890411	1
GSM1608663	1.263013699	0
GSM1608664	3.852054795	0
GSM1608665	3.64109589	1
GSM1608666	3.210958904	0
GSM1608667	0.449315068	0
GSM1608668	1	1
GSM1608669	7.079452055	0
GSM1608670	7.175342466	0
GSM1608671	1.093150685	1
GSM1608672	15.15068493	0
GSM1608673	16.70410959	0
GSM1608674	3.594520548	0
GSM1608675	0.328767123	1
GSM1608676	0.931506849	0

GSM1608677	1.175342466	1
GSM1608678	17.67945205	0
GSM1608679	3.597260274	0
GSM1608680	0.764383562	1
GSM1608681	3.046575342	0
GSM1608682	0.304109589	0
GSM1608683	0.534246575	1
GSM1608684	2.010958904	0
GSM1608685	1.484931507	1
GSM1608686	0.224657534	1
GSM1608687	0.830136986	1
GSM1608688	0.249315068	1
GSM1608689	0.139726027	0
GSM1608690	1.246575342	1
GSM1608691	0.194520548	0
GSM1608692	1.161643836	0
GSM1608693	1.57260274	1
GSM1608694	2.81369863	0
GSM1608695	15.43013699	0
GSM1608696	3.915068493	0
GSM1608697	0.830136986	1
GSM1608698	0.912328767	0
GSM1608699	0.224657534	1
GSM1608700	1.350684932	1
GSM1608701	0.465753425	0
GSM1608702	0.980821918	0
GSM1608703	6.134246575	0
GSM1608704	0.345205479	1
GSM1608705	6.353424658	0
GSM1608706	1.936986301	1
GSM1608707	3.630136986	1
GSM1608708	0.375342466	1
GSM1608709	3.923287671	1
GSM1608710	0.016438356	1
GSM1608711	0.432876712	0
GSM1608712	1.057534247	1
GSM1608713	1.78630137	0
GSM1608714	13.71232877	0
GSM1608715	0.509589041	0
GSM1608716	0.243835616	0
GSM1608717	1.210958904	1
GSM1608718	1.780821918	1
GSM1608719	0.84109589	0
GSM1608720	12.19178082	0
GSM1608721	0.515068493	1
GSM1608722	3.347945205	1
GSM1608723	3.37260274	0
GSM1608724	1.706849315	1
GSM1608725	0.539726027	1
GSM1608726	0.871232877	0
GSM1608727	1.04109589	1

GSM1608728	5.238356164	0
GSM1608729	1.150684932	0
GSM1608730	1.44109589	1
GSM1608731	0.580821918	0
GSM1608732	2.410958904	0
GSM1608733	1.02739726	1
GSM1608734	0.717808219	1
GSM1608735	0.153424658	1
GSM1608736	0.975342466	1
GSM1608737	1.304109589	1
GSM1608739	1.747945205	1
GSM1608740	1.191780822	1
GSM1608741	0.117808219	0
GSM1608742	0.115068493	1
GSM1608743	0.923287671	1
GSM1608744	1.073972603	1
GSM1608745	1.624657534	1
GSM1608746	2.621917808	1
GSM1608747	1.857534247	0
GSM1608748	0.887671233	0
GSM1608749	1.736986301	1
GSM1608750	1.77260274	0
GSM1608751	2.005479452	1
GSM1608752	0.690410959	0
GSM1608753	1.126027397	1
GSM1608754	0.920547945	1
GSM1608755	0.361643836	1
GSM1608756	2.35890411	0
GSM1608757	4.873972603	0
GSM1608758	5.364383562	1
GSM1608759	0.254794521	1
GSM1608760	0.197260274	1
GSM1608761	1.263013699	1
GSM1608762	1.249315068	0
GSM1608763	5.95890411	0
GSM1608764	7.671232877	0
GSM1608765	0.180821918	1
GSM1608766	0.84109589	0
GSM1608767	0.120547945	1
GSM1608768	2.835616438	0
GSM1608769	0.531506849	0
GSM1608770	9.21369863	0
GSM1608771	0.446575342	1
GSM1608772	1.432876712	0
GSM1608773	1.4	0
GSM1608774	1.126027397	1
GSM1608775	0.232876712	1
GSM1608777	1.868493151	1
GSM1608778	1.723287671	1
GSM1608779	0.452054795	1
GSM1608780	0.517808219	1

GSM1608781	0.180821918	0
GSM1608782	4.052054795	0
GSM1608783	0.175342466	1
GSM1608784	0.476712329	0
GSM1608785	3.238356164	1
GSM1608786	3.432876712	1
GSM1608787	1.876712329	0
GSM1608788	1.345205479	1
GSM1608789	2.016438356	1
GSM1608790	3.005479452	1
GSM1608791	2.161643836	0
GSM1608792	1.421917808	0
GSM1608793	4.887671233	0
GSM1608794	1.550684932	0
GSM1608795	3.778082192	0
GSM1608796	2.331506849	0
GSM1608798	0.843835616	0
GSM1608799	7.917808219	0
GSM1608800	1.747945205	1
GSM1608801	0.808219178	1
GSM1608802	0.421917808	1
GSM1608803	0.531506849	1
GSM1608804	0.397260274	1
GSM1608805	3.271232877	0
GSM1608806	4.084931507	0

2. Supplementary Figures

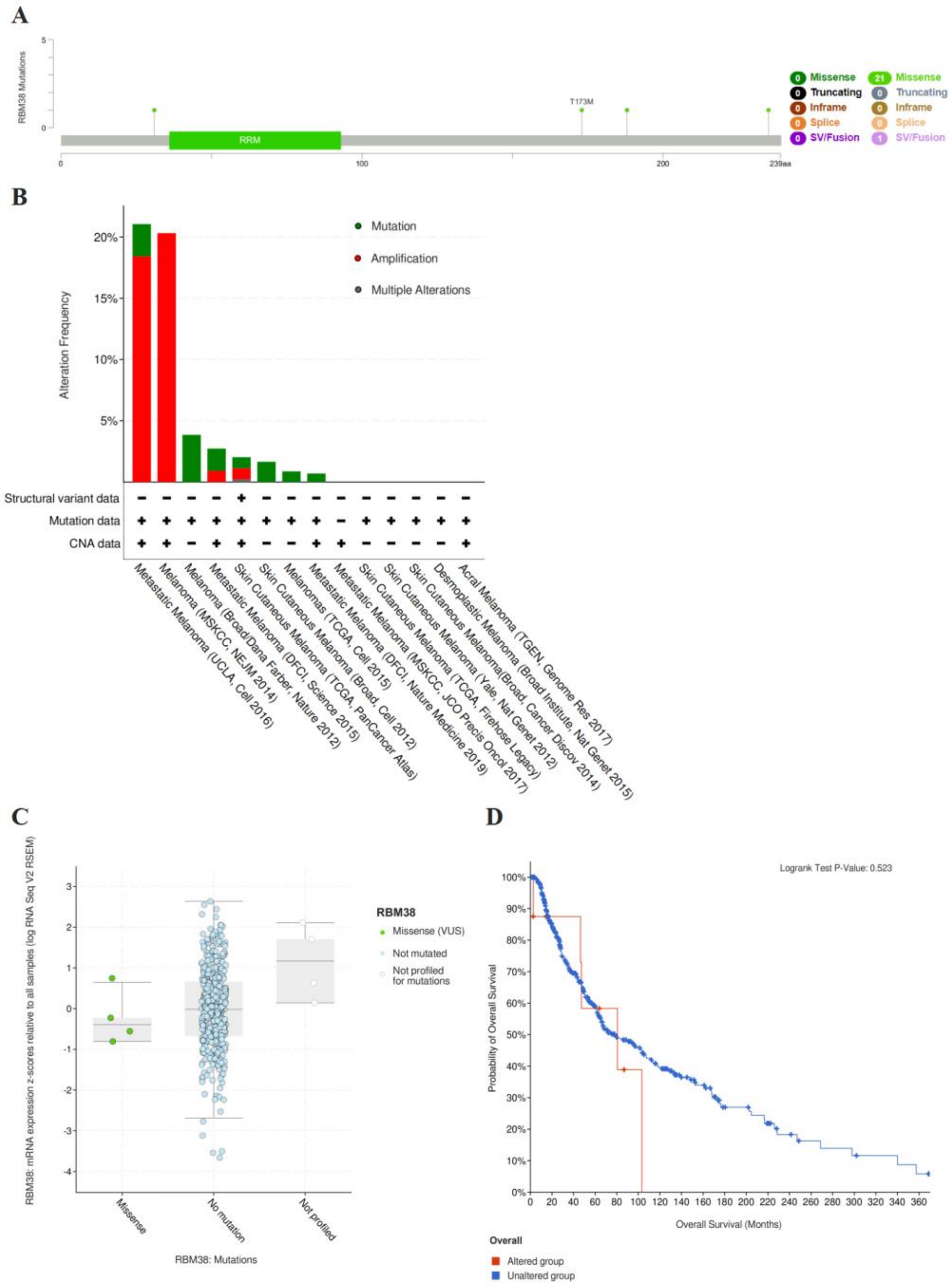

Figure S1. Mutation feature of RBM38 in melanoma. We analyzed the mutation features of RBM38 for melanoma using the cBioPortal tool. (**A**) The alteration frequency with mutation site are displayed. (**B**) The alteration frequency with mutation type are displayed. (**C**) The correlation between RBM38 and mutation types. (**D**) Overall survival analysis of the altered and unaltered group.

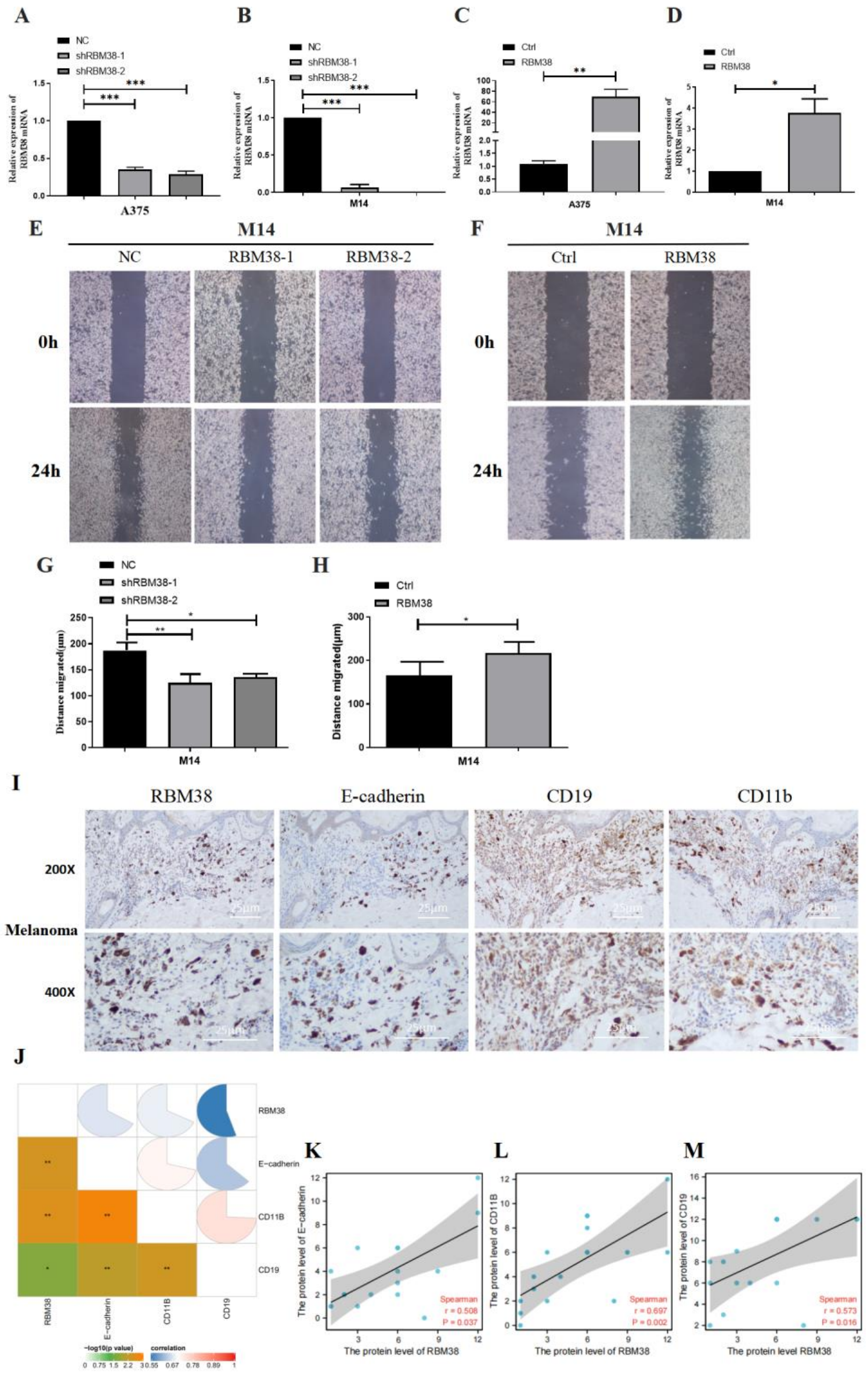

Figure S2. | RBM38 promoted the proliferation of malignant melanoma cells *in vitro*. (**A-D**) RT-qPCR analysis was used to confirm RBM38 overexpression and knockdown after transfection with lentivirus in A375 and M14 cell lines. (Data are shown as the mean ± SD of three replicates. **P* < 0.05, ***P* < 0.01, *** *P* < 0.001 by ANOVA test.) (**E-H**) Effects of RBM38 knockdown and overexpression in M14 cells by wound healing assay on cell migration. (Data are expressed as the mean ± standard deviation of three replicates). (**I**) Representative images of RBM38, CD19, CD11b, and E-cadherin staining in melanoma tissues (n = 17) by IHC staining. Scale bars indicated 25 μm. And RBM38 is correlated with CD19, CD11b, and E-cadherin. (**J**) The pheatmap of the correlation of RBM38, CD11b, CD19, and E-cadherin of Immunohistochemical staining analysis. (**P*<0.05, ***P*<0.01, ****P*<0.001), And RBM38 is

correlated with CD19, CD11b, and E-cadherin. (**K**) The correlation between RBM38 and E-cadherin of Immunohistochemical staining analysis. (**L**) The correlation between RBM38 and CD11b of Immunohistochemical staining analysis. (**M**) The correlation between RBM38 and CD19 of Immunohistochemical staining analysis. (For statistical comparison between two independent experimental groups (Student's t-test) and among more than two experimental groups (ANOVA test), appropriated statistical tests were assayed. *P<0.05, **P<0.01, ***P<0.001.)

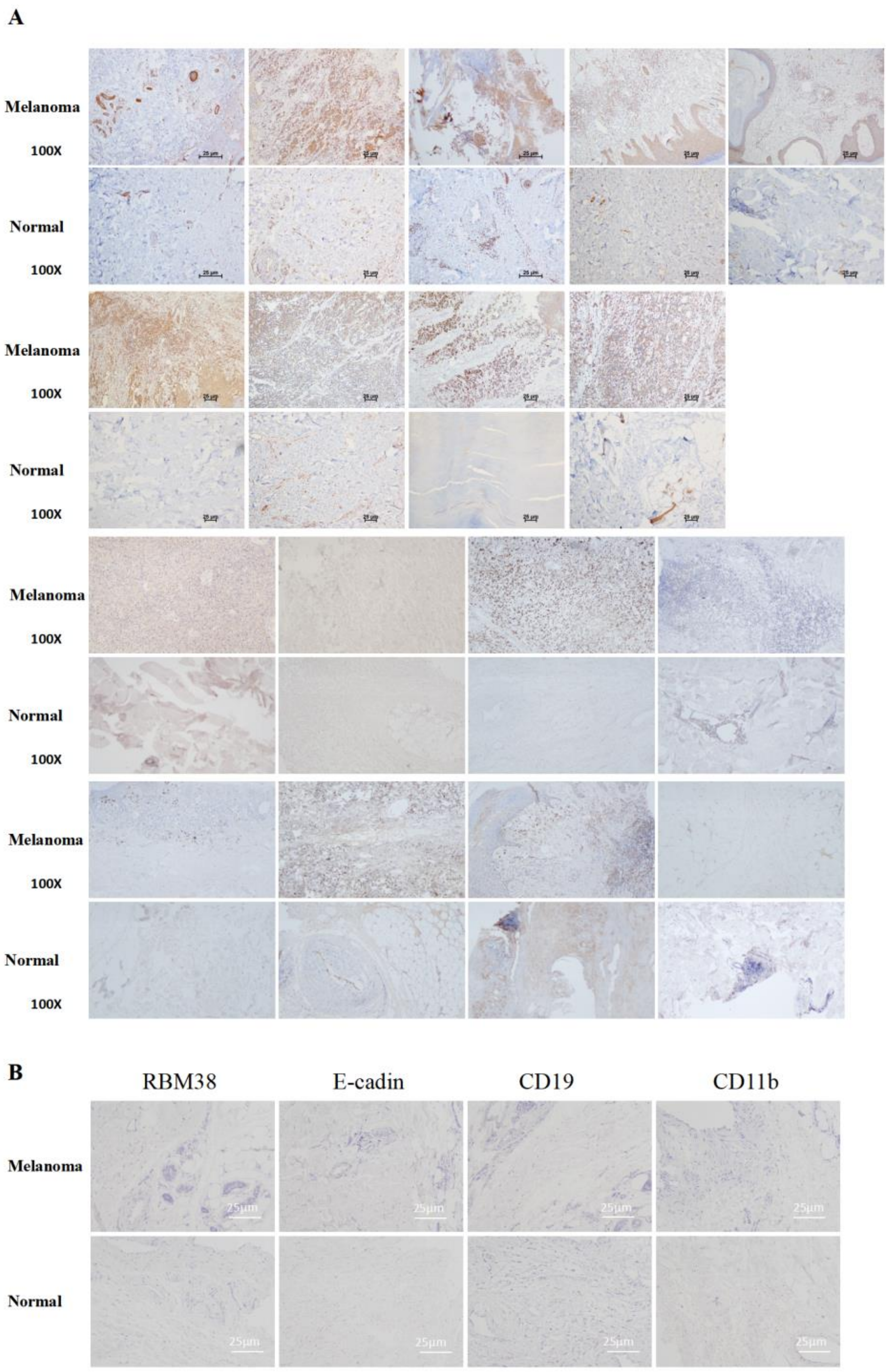

Figure S3 Representative images of RBM38, CD19, CD11b, and E-cadherin staining. (**A**) Other 17 paired images of RBM38 staining in melanoma and normal tissues. (**B**) Representative images of negative control of tumor tissues and normal tissues staining.

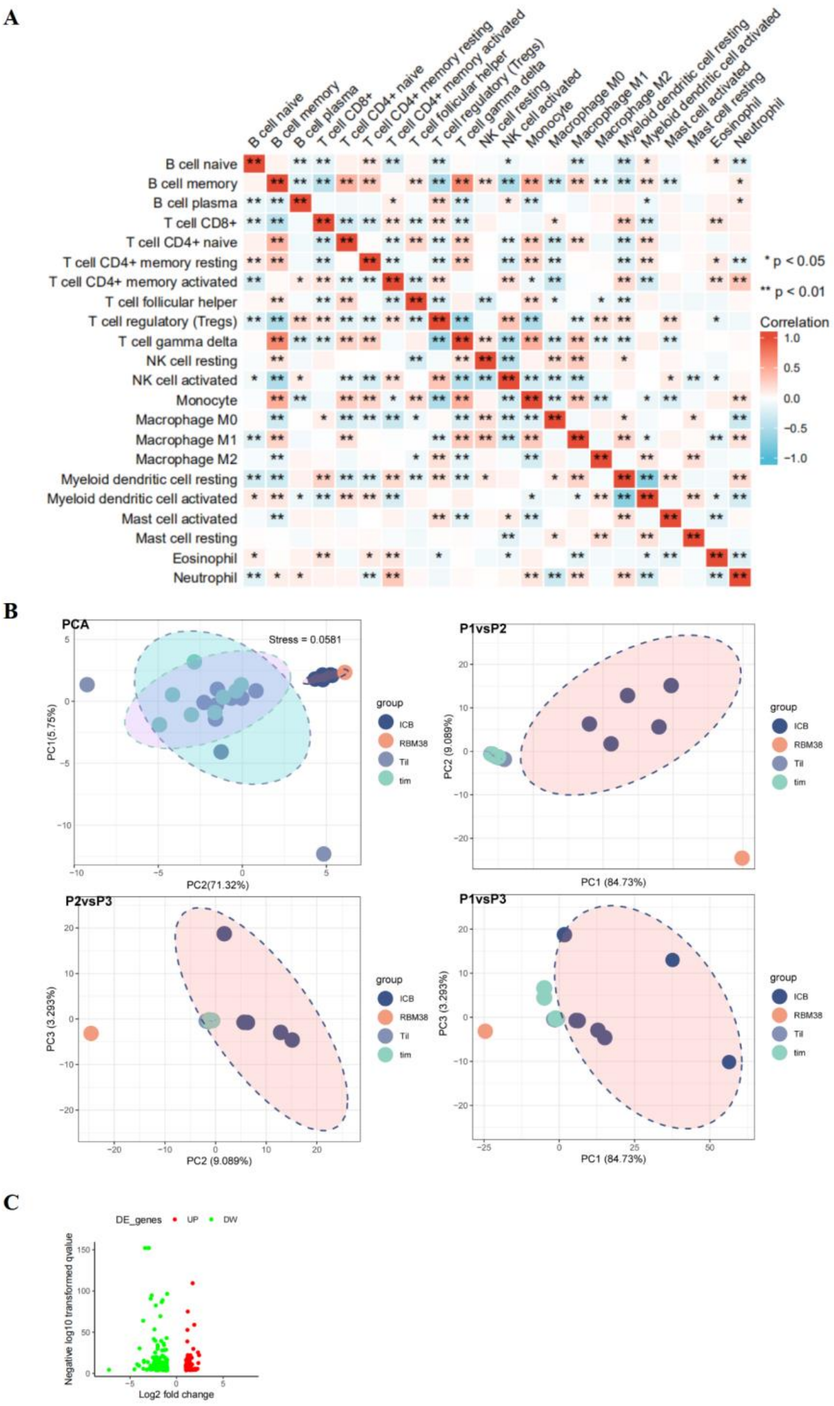

Figure S4. The relevance of immune cells in the TME and the correlation of TiL / TiM, ICB, and RBM38. (**A**) The immune cells exhibited intricate relevance among each other in the TME(**P*<0.05, ***P*<0.01, ****P*<0.001). (**B**) The results showed RBM38 is correlated with ICB and not correlated with TiL and TiM through PCA analysis. (**C**) Total of 158 downregulated genes were differentially identified from mRNA sequencing.

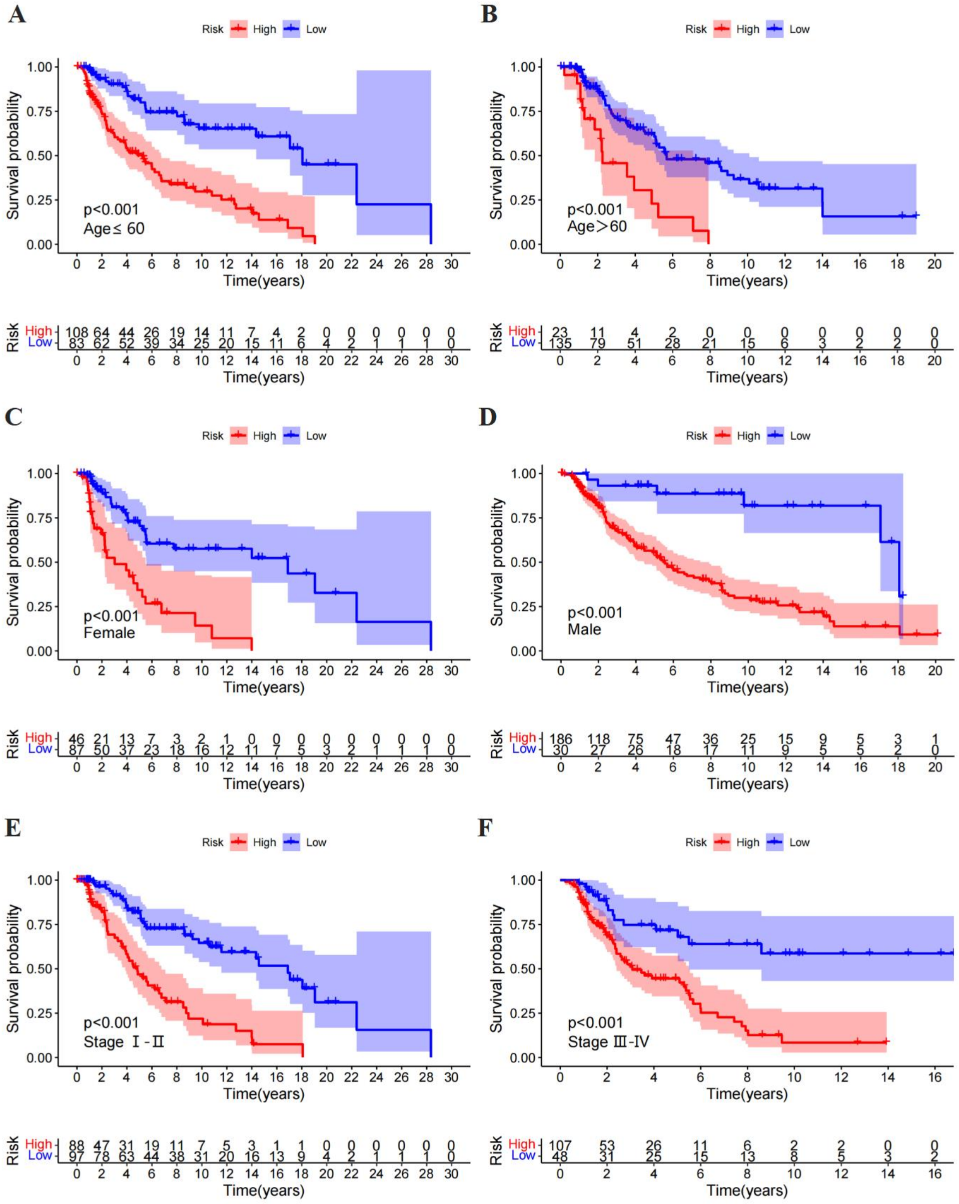

Figure S5. The OS of the low- and high-risk subgroups separated by clinicopathological parameters in the training group. (**A-B**) In the training group, the OS of the low- and high-risk subgroups separated by age were assessed. (**C-D**) In the training group, the OS of the low- and high-risk subgroups separated by gender were assessed. (**E-F**) In the training group, the OS of the low- and high-risk subgroups separated by stage were assessed.

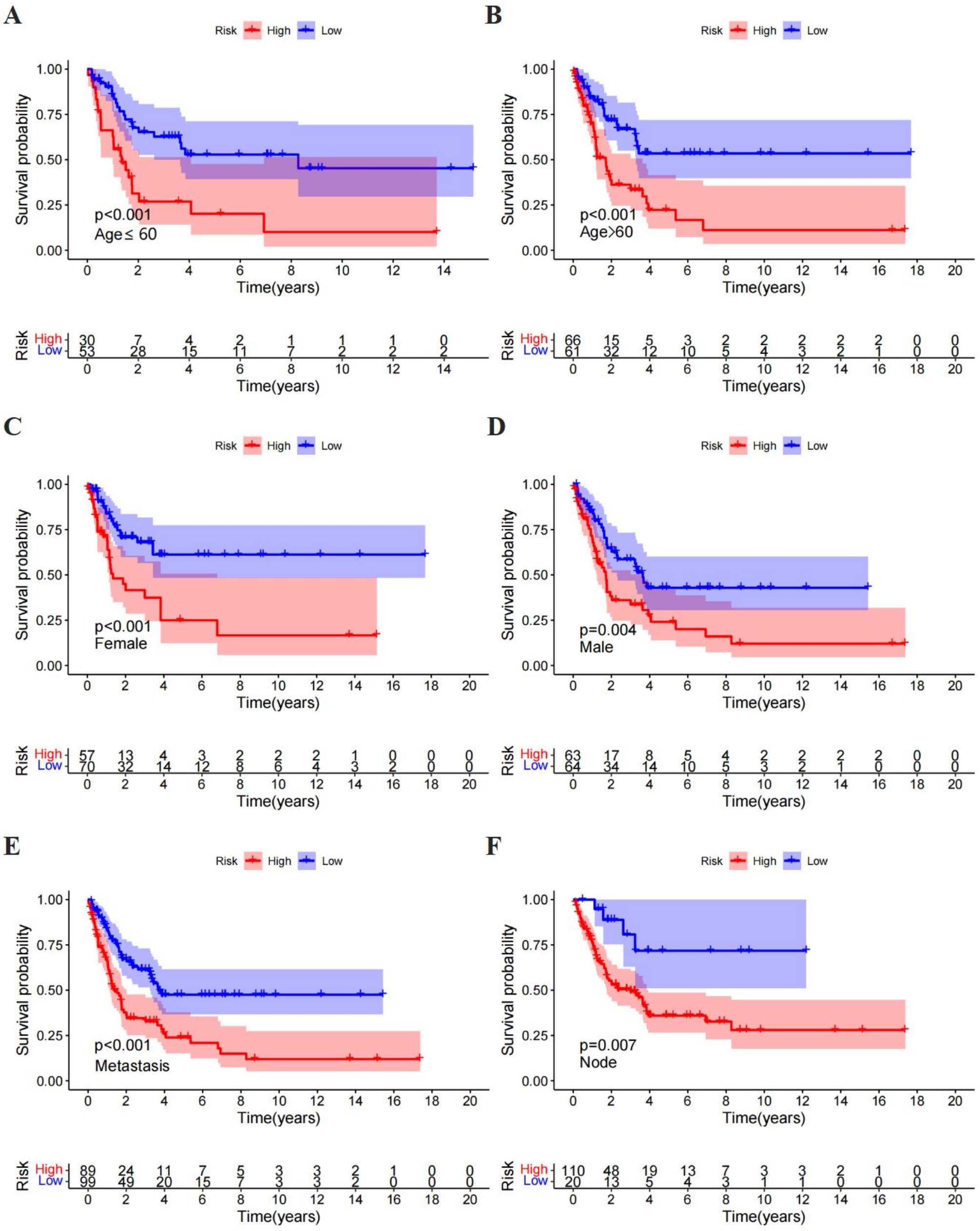

Figure S6. The OS of the low- and high-risk subgroups separated by clinicopathological parameters in the validation group. (**A-B**) In the validation group, the OS of the low- and high-risk subgroups separated by age were assessed. (**C-D**) In the validation group, the OS of the low- and high-risk subgroups separated by gender were assessed. (**E-F**) In the validation group, the OS of the low- and high-risk subgroups separated by metastasis and node were assessed.

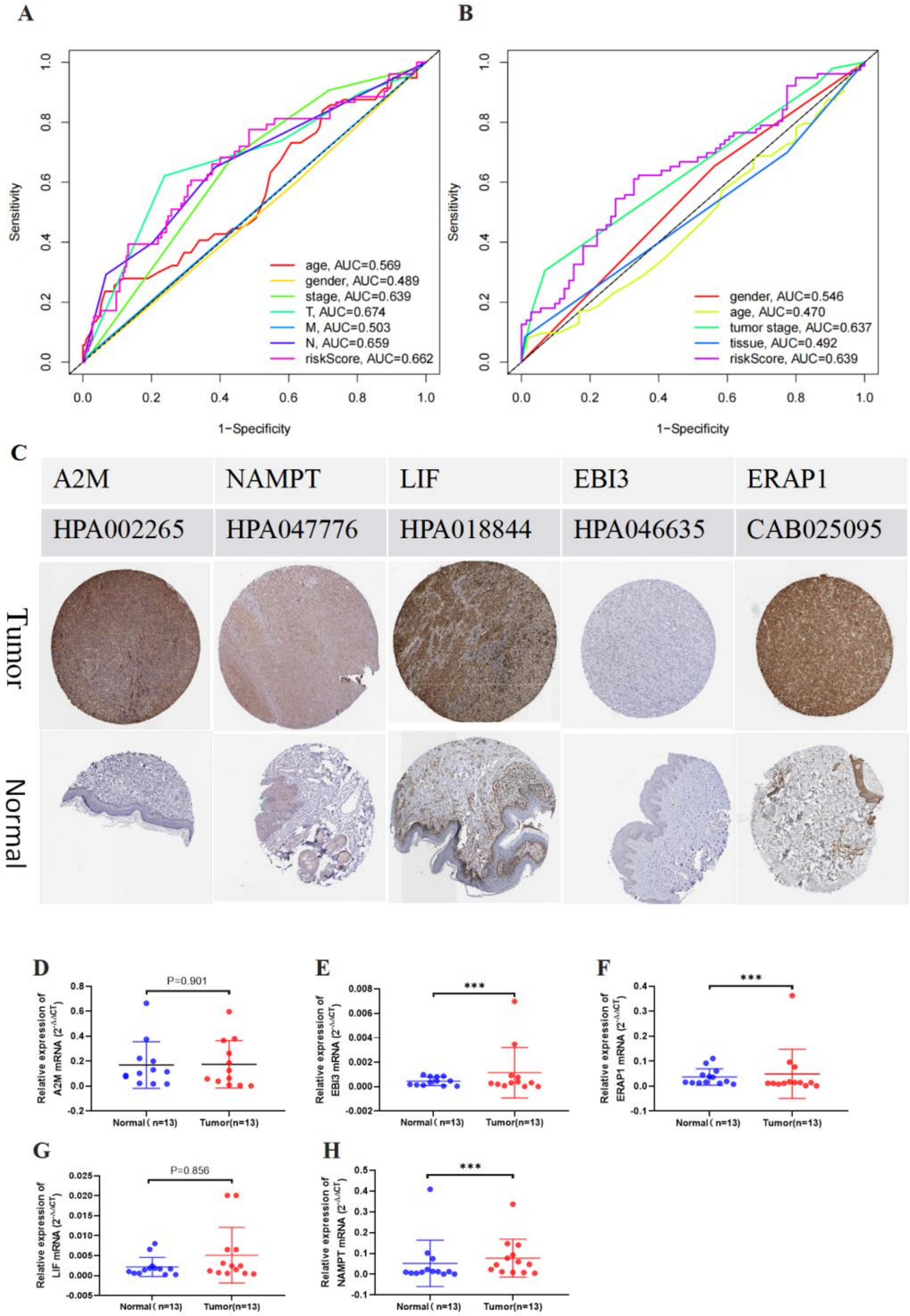

Figure S7. Validation of the five-gene signature and immunohistochemical assay. (**A-B**) In the training and validation cohort, risk score, age, gender, clinical stage, tumor, metastasis, and AUC value of nodes with clinical information were explored. (**C**) Protein expression of five hub genes (A2M, NAMPT, LIF, EBI3, and ERAP1) was assessed by an immunohistochemical assay in malignant melanoma, according to the Human Protein Atlas website. (**D-H**) RT-qPCR analysis of A2M, EBI3, NAMPT, LIF, and ERAP1 expressions of mRNA in 13 paired melanoma tissues and matched normal tissues quantified after transfection. (Data are shown as the mean ± SD of three replicates. **P* < 0.05, ***P* < 0.01, *** *P* < 0.001 by ANOVA test.)
